# Targeting Ferroptosis Pathways for Synaptic Protection in Sevoflurane‐Induced Cognitive Impairment: A Nanomedicine Approach

**DOI:** 10.1002/cns.70850

**Published:** 2026-03-31

**Authors:** Bin Zhao, Changming Wang, Mingming Zhang

**Affiliations:** ^1^ Department of Anesthesiology People's Hospital of China Medical University (People's Hospital of Liaoning Province) Shenyang China; ^2^ Department of Anesthesiology, School and Hospital of Stomatology China Medical University Shenyang China

**Keywords:** ferroptosis, nanodrug delivery system, postoperative cognitive dysfunction, sevoflurane, synaptic plasticity

## Abstract

**Background:**

Postoperative cognitive dysfunction (POCD) is an increasingly recognized neurological complication following surgery, particularly in elderly patients. It significantly hinders recovery and impairs the quality of life. Sevoflurane, a commonly used volatile anesthetic, has been implicated in enhancing the incidence and severity of POCD. At the molecular level, synaptic dysfunction is a major contributor to cognitive decline associated with POCD. Recent studies have highlighted ferroptosis, an iron‐dependent form of cell death driven by lipid peroxidation, as a critical mechanism behind synaptic damage and cognitive decline.

**Methods:**

This review synthesizes current research on the role of ferroptosis in POCD, focusing on its impact on synaptic dysfunction. We also explore the potential of nanomedicine, particularly intelligent responsive nanodrug delivery systems, in targeting ferroptotic pathways. These nanoplatforms, with their high brain delivery efficiency and specificity, have been shown to modulate ferroptosis signaling and reduce neuronal injury, thereby potentially promoting cognitive recovery.

**Results:**

Ferroptosis plays a significant role in exacerbating cognitive deficits by disrupting synaptic membranes and mitochondria, which contribute to synaptic dysfunction. Emerging evidence suggests that interventions targeting ferroptosis pathways can mitigate these effects, offering a novel therapeutic avenue for POCD. Nanomedicine approaches, especially those utilizing responsive nanodrug delivery systems, have shown promise in effectively targeting ferroptotic pathways with high specificity, leading to reductions in synaptic injury and enhanced cognitive recovery.

**Conclusion:**

Ferroptosis is a key mechanism driving synaptic dysfunction and cognitive decline in POCD. Targeting ferroptotic pathways using nanomedicine‐based strategies holds considerable promise for mitigating POCD and promoting cognitive recovery. Further research is needed to optimize these therapeutic approaches for clinical application in POCD management.

## Graphical abstract

Sevoflurane exposure triggers iron‐dependent lipid peroxidation and ferroptosis, leading to synaptic damage and cognitive dysfunction. Targeted nanodelivery systems that cross the blood–brain barrier can precisely modulate ferroptotic pathways, reduce oxidative injury, and rescue synaptic integrity, offering a promising strategy for preventing postoperative cognitive dysfunction.

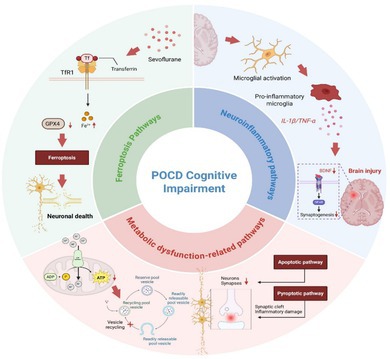

**Abbreviations**
AA: arachidonic acid
AAV: adeno‐associated virus
ACSL4: acyl‐CoA synthetase long‐chain family member 4
AdA: adrenic acid
AI: artificial intelligence
ALOX15: arachidonate 15‐lipoxygenase
APP: amyloid precursor protein
Arc: activity‐regulated cytoskeleton‐associated protein
BBB: blood–brain barrier
BDNF: brain‐derived neurotrophic factor
CAT: catalase
CPPs: cell‐penetrating peptides
DG: dentate gyrus
DMN: default mode network
ER: endoplasmic reticulum
Fer‐1: ferrostatin‐1
FPN1: ferroportin 1
FTH1: ferritin heavy chain 1
GDNF: glial cell line‐derived neurotrophic factor
GPX4: glutathione peroxidase 4
GSH: glutathione
GSH‐Px: glutathione peroxidase
IADL: Instrumental Activities of Daily Living
IL‐1β: interleukin‐1β
iPSCs: induced pluripotent stem cells
ISPOCD: International Study of Postoperative Cognitive Dysfunction
LIP: labile iron pool
LOX: lipoxygenases
LTP: long‐term potentiation
MDA: malondialdehyde
MMP‐9: matrix metalloproteinase‐9
MMSE: Mini‐Mental State Examination
MoCA: Montreal Cognitive Assessment
NPs: nanoparticles
NSC‐Exo: neural stem cell‐derived exosomes
PCL: polycaprolactone
PEG: polyethylene glycol
PEI: polyethyleneimine
PLA: polylactic acid
PLGA: poly(lactic‐co‐glycolic acid)
POCD: postoperative cognitive dysfunction
PSDs: postsynaptic densities
PSP: postsynaptic potentials
PUFAs: polyunsaturated fatty acids
ROS: reactive oxygen species
RVG: rabies virus glycoprotein
scRNA‐seq: single‐cell RNA sequencing
SIRT1: sirtuin 1
SOD: superoxide dismutase
TEM: transmission electron microscopy
TfR: transferrin receptor
TfR1: transferrin receptor 1
TMS: transcranial magnetic stimulation
TNF‐α: tumor necrosis factor‐α

## 1 Introduction

Sevoflurane, one of the most widely used inhalational anesthetics in clinical practice, is favored for its rapid induction, quick metabolism, and excellent analgesic properties, and is extensively applied in pediatric, geriatric, and neurosurgical settings [1]. However, recent clinical and preclinical studies have indicated that sevoflurane anesthesia may trigger postoperative cognitive dysfunction (POCD), with older patients appearing to be at particularly elevated risk [2]. Similar findings have also been reported in developmental models [3]. Consistent findings from both clinical and animal studies support this association: exposure to sevoflurane leads to cognitive deficits in elderly individuals and juvenile animal models, manifesting as marked impairments in attention, memory, and executive function [3, 4]. The pathophysiological basis of POCD is multifaceted. While earlier research primarily focused on classical pathways such as neuroinflammation and oxidative stress, recent studies have shifted attention to synaptic plasticity impairment as a more direct phenotypic mechanism underlying cognitive decline [5]. Moreover, ferroptosis—an emerging form of programmed cell death—has been implicated in this process. Evidence suggests that sevoflurane anesthesia induces iron‐dependent lipid peroxidation, thereby compromising the integrity and function of synaptic membranes [6].

The stability and functional integrity of synaptic structures form the foundation of learning and memory processes. Recent studies have shown that repeated exposure of neonatal mice to sevoflurane significantly suppresses adult hippocampal neurogenesis, accompanied by reductions in synaptic plasticity‐related proteins such as sirtuin 1 (SIRT1), BDNF, and activity‐regulated cytoskeleton‐associated protein (Arc), as well as decreased long‐term potentiation (LTP), ultimately resulting in sustained impairments in learning and memory during adulthood [3]. Existing evidence further demonstrates that an enriched environment can alleviate sevoflurane‐induced impairments in hippocampal synaptic plasticity and cognitive function in neonates, reinforcing the notion that synaptic structures are key targets of anesthesia‐induced learning and memory deficits [7]. Meanwhile, ferroptosis, a recently recognized form of programmed cell death characterized by iron‐dependent lipid peroxidation, has been identified as a critical mechanism linking metabolic imbalance to synaptic membrane damage [6, 8]. In a recent study, sevoflurane was found to upregulate PRKCD, thereby inhibiting the Hippo signaling pathway and activating ferroptosis, leading to hippocampal neuronal damage and cognitive impairment; these effects were reversed by PRKCD knockout [9]. In addition, both rats and primary hippocampal neurons exposed to sevoflurane exhibited features of iron overload, including increased iron ion levels and downregulation of GPX4 and SLC7A11. Activation of endoplasmic reticulum (ER) stress via ATF3 contributed to lipid peroxidation and subsequent cell death, ultimately resulting in elevated ferroptosis rates in neonatal hippocampal neurons—effects that were alleviated by iron chelators in rats [10].

Precise modulation targeting ferroptosis has provided a novel breakthrough for the intervention of POCD. A study in rodents revealed that ferroptosis markers in hippocampal tissue—such as upregulated PRKCD and downregulated GPX4/SLC7A11—were significantly activated following sevoflurane treatment, accompanied by marked cognitive impairments [9]. However, traditional small‐molecule interventions have faced major challenges, including limited permeability across the blood–brain barrier (BBB), systemic toxicity, and low targeting efficiency, which have substantially hindered the intracerebral application of ferroptosis inhibitors [11]. Nanodrug delivery systems, owing to their favorable brain‐targeting properties, biocompatibility, and intelligent responsiveness, have demonstrated distinct advantages in overcoming physiological barriers and achieving spatial–temporal control of drug delivery [11]. With customizable targeting abilities, environmental responsiveness (e.g., to reactive oxygen species [ROS] and pH), and excellent biocompatibility, these systems have emerged as ideal platforms for BBB penetration and controlled delivery [11]. Recent studies have developed nanoplatforms based on liposomes, poly(lactic‐co‐glycolic acid) (PLGA), biomimetic membranes, and exosomes to successfully deliver ferroptosis inhibitors such as Ferrostatin‐1 (Fer‐1) and Liproxstatin‐1, thereby enhancing neuroprotection [11, 12]. For instance, RVG29‐modified targeted lipid nanoparticles (NPs) successfully transported Fer‐1 across the BBB and improved its neuroprotective efficacy in a stroke model [12]. Similarly, calcium carbonate‐mineralized liposomal (CLF) carriers encapsulating Fer‐1 enabled precise delivery to lesioned neural regions and mitigated ferroptosis‐induced neuronal injury by delaying drug release and suppressing lipid peroxidation [11]. Notably, when combined with ROS/pH‐responsive systems, such nanoplatforms demonstrated synergistic features of lesion‐specific targeting and controlled release.

Although substantial progress has been made in elucidating the mechanisms underlying POCD, current reviews still exhibit two major limitations. First, many studies focus narrowly on ferroptosis as a single mechanism, overlooking parallel pathways such as neuroinflammation and metabolic dysfunction, which may act as the predominant drivers in subsets of patients [13]. Second, evidence from multisystem injury models indicates that interventions targeting only one pathway often fail to simultaneously modulate ferroptosis, neuroinflammation, oxidative stress, and other co‐occurring pathological processes [14]. Given that neuroinflammation is widely observed in POCD [15], inhibiting ferroptosis alone is unlikely to yield optimal cognitive improvement. Therefore, it is necessary to establish an integrated framework—based on “multiple parallel mechanisms and multi‐target intervention”—to clarify the interactions and relative contributions of ferroptosis and other pathogenic pathways, thereby improving the clinical relevance of mechanistic interpretations.

This review aims to address this gap by constructing a structured and integrative model of the POCD mechanistic chain. It systematically consolidates the triadic framework of ferroptosis, synaptic plasticity, and intelligent nanointerventions to establish a structured logical framework for POCD intervention research across three interconnected dimensions: network pathology, delivery strategies, and translational challenges. Through this framework, this review builds a structured logic model for POCD‐targeted intervention, synthesizes the current body of evidence and key bottlenecks, and proposes coordinated directions for future translational exploration.

## 2 Sevoflurane‐Induced Synaptic Plasticity Impairment and Cognitive Dysfunction Mechanisms

### 2.1 Sevoflurane‐Induced Synaptic Structural‐Functional Network Disruption and Multimechanistic Damage

Synaptic plasticity, the fundamental basis for learning and memory formation, depends on the coordinated maintenance of synaptic structural integrity, vesicle recycling efficiency, and neural network connectivity density [16]. Sevoflurane anesthesia has been shown to impair synaptic function at both structural and functional levels. Structurally, exposure to sevoflurane induces widening of the synaptic cleft, thinning of the postsynaptic density (PSD), and a reduction in synaptic vesicle number in the hippocampal CA1 region and prefrontal cortex. These changes are accompanied by the downregulation of key synaptic proteins such as PSD‐95, Synapsin I, and Homer1 [9, 17]. Functionally, sevoflurane disrupts vesicle recycling and neurotransmitter release systems by inhibiting Rab3a and Synaptophysin, resulting in reduced presynaptic transmission efficiency. This is reflected in decreased LTP amplitudes in electrophysiological recordings, manifested as impaired action potential‐evoked transmission, weakened synaptic strength, and unstable network connectivity [5, 18, 19].

**FIGURE 1:**
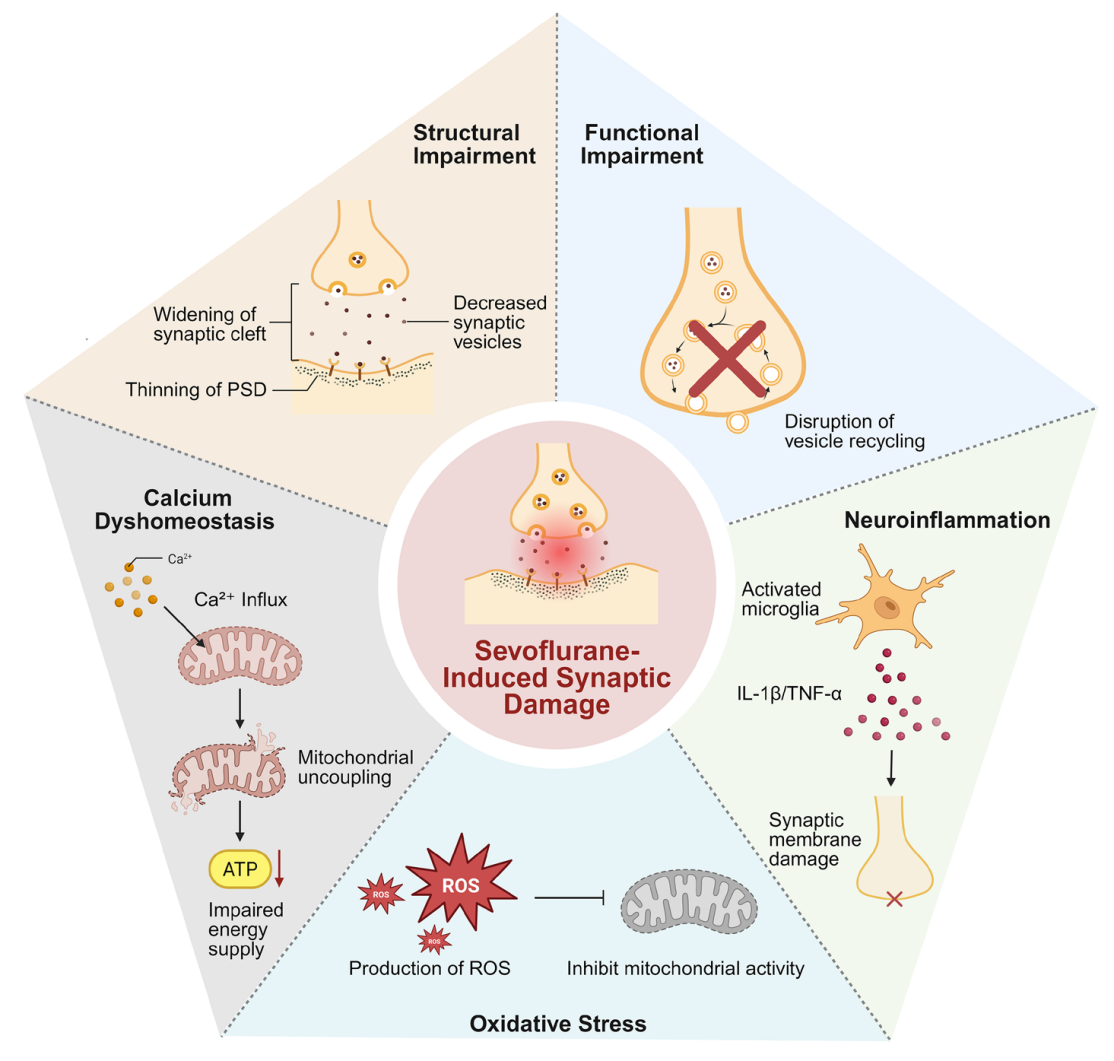
Multi‐mechanistic integration of sevoflurane‐induced synaptic injury. Synaptic injury caused by sevoflurane exposure involves five interrelated pathological processes: Structural disruption (widening of the synaptic cleft, reduced synaptic vesicles, and thinning of the PSD); functional impairment (impaired vesicle recycling); calcium homeostasis disturbance (Ca^2+^ overload and mitochondrial uncoupling); neuroinflammation (microglial activation and pro‐inflammatory cytokine release); and oxidative stress (excessive ROS production leading to mitochondrial dysfunction). ATP, adenosine triphosphate; IL‐1β, interleukin‐1β; PSD, postsynaptic density; ROS, reactive oxygen species; TNF‐α, tumor necrosis factor‐α.

Notably, this series of synaptic structural and functional impairments does not occur in isolation but results from the combined effects of oxidative stress and neuroinflammation [20, 21]. Sevoflurane exposure induces excessive generation of ROS, inhibits mitochondrial complex activity, and damages synaptic membrane lipids and proteins, leading to the accumulation of malondialdehyde (MDA) and downregulation of antioxidant enzymes such as superoxide dismutase (SOD) and glutathione peroxidase (GSH‐Px). Concurrently, calcium overload and mitochondrial uncoupling further compromise energy supply and the maintenance of synaptic excitability [22]. In addition, microglial activation and the subsequent release of proinflammatory cytokines, including interleukin‐1β (IL‐1β) and tumor necrosis factor‐α (TNF‐α), not only exacerbate synaptic membrane injury but also impair synaptic repair capacity by disrupting neurotrophic signaling pathways, such as the brain‐derived neurotrophic factor (BDNF)–TrkB axis (Figure 1) [23].

Brain imaging studies have shown that sevoflurane markedly suppresses functional connectivity between the hippocampus and cortical regions and disrupts activity within the default mode network (DMN), indicating impaired synaptic network integration following anesthetic exposure [24]. Moreover, dynamic alterations in DMN connectivity have also been observed in inflammation‐induced cognitive impairment models [25], further supporting a link between network‐level dysfunction and cognitive decline. Overall, these interacting mechanisms suggest that postoperative cognitive dysfunction arises from pathological changes extending from synaptic abnormalities at the protein level to large‐scale network disturbances, forming a key neurobiological foundation of POCD (Table S1).

### 2.2 Synaptic Vulnerability During Brain Development and Aging

Sevoflurane‐induced impairment of synaptic plasticity exhibits significant spatiotemporal susceptibility across different stages of the lifespan. During brain development, which represents a critical window for synaptogenesis, pruning, and network formation, sevoflurane exposure exerts profound effects on neural circuit maturation [26, 27, 28]. Studies have shown that neonatal mice exposed to sevoflurane between postnatal days 7 and 14 exhibit marked impairments in learning and memory during adulthood, along with downregulation of hippocampal synaptic marker proteins such as PSD‐95 and BDNF [3].

MicroRNAs that regulate dendritic spine formation and synaptic plasticity, such as miR‐132, play crucial roles during brain development [29]. Sevoflurane has been shown to reduce dendritic spine density [5], suggesting that the developing brain may be particularly sensitive to sevoflurane at the level of microRNA‐mediated regulatory pathways. Meanwhile, sevoflurane may also exert long‐term effects on neural developmental pathways through epigenetic mechanisms, such as altered DNA methylation levels, implying its potential to reshape gene expression profiles and interfere with neuroplasticity programs over extended periods [30].

In the aging brain, synaptic connectivity is already diminished due to physiological aging, and the decline in cognitive reserve significantly reduces tolerance to anesthetic agents [31, 32]. Studies have shown that aged mice exposed to sevoflurane exhibit a marked reduction in postsynaptic potentials (PSPs) and impaired induction of LTP [33]. Moreover, sevoflurane has been reported to promote Tau protein phosphorylation and dendritic spine loss, thereby exacerbating synaptic injury [34, 35]. Such findings suggest that the aged brain possesses limited capacity for self‐repair following synaptic injury and exhibits elevated baseline levels of inflammation and oxidative stress, which collectively exacerbate the neurotoxicity of sevoflurane [33].

Together, these studies indicate that both the developing and aging brain exhibit intrinsic vulnerabilities in synaptic plasticity regulation, constituting a “susceptibility window” for sevoflurane‐related cognitive dysfunction. In clinical practice, the immaturity of neural development in pediatric patients and the diminished brain reserve in elderly individuals necessitate enhanced risk assessment and tailored intervention strategies when formulating anesthetic plans [36, 37]. Future research should therefore focus on identifying individualized intervention windows and validating mechanistic targets across different life stages, thereby laying the groundwork for precision prevention and treatment of POCD (Figure S1).

Moreover, both developmental and aging stages share a common susceptibility mechanism characterized by complement system overactivation–mediated synaptic loss: (1) Developmental stage: Exposure to sevoflurane upregulates complement components C1qa and C3, activating microglia‐mediated pathological synaptic pruning, which markedly reduces synaptic density during the peak of synaptogenesis [38]. Normally, such pruning occurs during the physiological “synaptic elimination phase,” but sevoflurane accelerates complement release through the NF‐κB pathway, leading to premature and excessive pruning [39]. (2) Aging stage: In aged mice (24 months old), sevoflurane anesthesia increases C1qa expression in the hippocampus, enhances the phagocytic activity of microglia toward synapses, and shifts pruning targets from redundant synapses to functional synapses [40]. Collectively, such overactivation of the complement–microglia axis results in synaptic network disconnection during both development (due to runaway pruning) and aging (due to misdirected pruning) (Figure S2).

## 3 Mechanistic Insights Into Ferroptosis Involvement in Synaptic Injury and Cognitive Impairment

### 3.1 The Molecular Mechanisms of Ferroptosis Involve Lipid Peroxidation, Glutathione Peroxidase 4 (GPX4) Inactivation, and Mitochondrial Dysfunction

Ferroptosis is a form of programmed cell death driven by iron‐dependent lipid peroxidation, mechanistically distinct from classical apoptosis, necrosis, and autophagy [41]. Its defining features include increased intracellular free iron, elevated levels of ROS, oxidation of polyunsaturated fatty acids (PUFAs) in cellular membranes, and functional loss of GPX4 [42, 43]. GPX4 is a critical antioxidant enzyme that prevents the propagation of lipid peroxidation chain reactions, and its inactivation represents a hallmark event in the initiation of ferroptosis [42]. Sevoflurane exposure was found to suppress GPX4 expression and promote the accumulation of lipid peroxidation products, thereby triggering ferroptotic pathways in neuronal cells [10].

Moreover, mitochondria also play a central role in ferroptosis [41]. Unlike classical necrotic processes, ferroptosis is not characterized by mitochondrial swelling; instead, it exhibits distinct morphological features, including reduced mitochondrial membrane potential, loss of cristae structure, and overall shrinkage of mitochondrial size [44, 45]. Studies have shown that in the brains of sevoflurane‐treated mice, abnormal mitochondrial morphology is accompanied by impaired oxidative phosphorylation, suggesting mitochondrial dysfunction and activation of ferroptosis‐related mechanisms [46]. Lipid peroxidation primarily targets PUFAs within mitochondrial membranes, leading to disruption of membrane integrity and membrane potential, ultimately resulting in energy metabolism collapse and cell death [43, 47]. In addition, enzymes such as lipoxygenases (LOX), acyl‐CoA synthetase long‐chain family member 4 (ACSL4), and arachidonate 15‐lipoxygenase (ALOX15) catalyze PUFA oxidation, thereby amplifying lipid peroxidation chain reactions—an essential enzymatic step in the ferroptotic process (Figure S3) [48].

### 3.2 Sevoflurane Disrupts Cerebral Iron Homeostasis and Induces Oxidative Stress

Iron homeostasis in the healthy nervous system is tightly regulated, as iron plays essential roles in neurotransmitter synthesis, myelin formation, and mitochondrial respiration [49]. However, excessive free iron can generate hydroxyl radicals via the Fenton reaction, thereby initiating lipid peroxidation and oxidative damage that ultimately triggers ferroptosis [50]. Studies have shown that exposure to sevoflurane can induce iron accumulation in brain tissue, accompanied by altered expression of key iron‐metabolism proteins, including downregulation of ferroportin 1 (FPN1) and transferrin receptor 1 (TfR1) and upregulation of ferritin, indicating a disruption of iron homeostasis [51].

Additionally, sevoflurane impairs the expression of FPN1, the principal iron export protein, resulting in intracellular iron accumulation [41]. Transcriptomic analyses together with validation experiments have demonstrated that sevoflurane exposure leads to upregulation of ACSL4 and downregulation of GPX4 and SLC7A11, indicating activation of ferroptosis‐related signaling pathways [52]. Sevoflurane‐induced oxidative stress and iron dysregulation appear to reinforce one another: elevations in ROS are accompanied by increased TfR1 expression and reduced FPN levels, thereby enhancing iron uptake and expanding the labile iron pool [41]. In summary, sevoflurane activates ferroptosis through a dual mechanism involving disrupted iron homeostasis and accumulated oxidative stress (Figure S4).

### 3.3 Ferroptosis Marker Molecules (ACSL4, SLC7A11, Ferritin Heavy Chain 1 [FTH1]) and Synaptic Dysfunction

Ferroptosis not only results in neuronal death but also specifically affects synaptic structure and function. Key marker molecules serve as critical mediators in this process [53]. ACSL4 is an essential enzyme that promotes the esterification of PUFAs, such as arachidonic acid (AA) and adrenic acid (AdA), rendering synaptic membranes particularly susceptible to oxidative damage [54]. ACSL4 is a key enzyme that determines the acylation of polyunsaturated fatty acids (PUFAs) and their susceptibility to oxidative damage [55]. Sevoflurane exposure has been shown to upregulate ACSL4 expression and increase the propensity for lipid peroxidation [46]. Such changes may lower the antioxidant threshold of synaptic structures and disrupt synaptic homeostasis and signal transmission.

SLC7A11 encodes the core subunit of system Xc^−^, which mediates cystine‐glutamate exchange and regulates glutathione (GSH) synthesis and cellular antioxidant defense [10]. Sevoflurane downregulates SLC7A11 expression, limiting GSH availability and indirectly leading to GPX4 inactivation and uncontrolled lipid peroxidation [56]. FTH1, the major subunit of ferritin, plays a critical role in sequestering iron, mitigating iron‐mediated toxicity, and suppressing ferroptosis [57]. In neurotoxic models, downregulation of FTH1 leads to an increase in free Fe^2+^ and enhanced lipid peroxidation, indicating that ferritin‐related signaling is closely linked to synaptic impairment. This mechanism may also contribute to sevoflurane‐induced synaptic dysfunction (Figure S5) [57].

The dynamic changes in these core ferroptosis‐related molecules are closely associated with synaptic function, suggesting their potential as early molecular indicators of synaptic injury (Table S2) [52].

Moreover, alterations in the expression of these ferroptosis‐associated markers not only reflect localized cellular damage responses but may also contribute to broader synaptic network dysfunction by affecting membrane stability, neurotransmitter synthesis and release, and regulation of glutamate excitotoxicity [50, 58]. Notably, under sevoflurane exposure, the spatial and temporal overlap between ferroptotic signaling and synaptic plasticity impairment indicates that ferroptosis may serve as a critical mechanistic bridge linking cellular stress to cognitive dysfunction [46, 52] (Table S3).

### 3.4 Crosstalk Between Ferroptosis, Synaptic Plasticity, and Cognitive Dysfunction

Activation of ferroptosis not only contributes to neuronal loss but also disrupts the fine architecture of synapses and may impair synaptic signal transmission, forming a mechanistic bridge in the development of POCD [46, 59]. On one hand, ferroptosis‐related lipid peroxidation and structural synaptic damage can interfere with glutamatergic signaling, thereby reducing synaptic plasticity [46]. On the other hand, iron overload‐induced calcium dyshomeostasis interferes with synaptic vesicle release and recycling, thereby compromising presynaptic function [60, 61].

Notably, ferroptosis activation observed in current experimental studies exhibits an acute profile, which is temporally misaligned with the delayed onset of clinical POCD—an inconsistency that has critical implications for mechanistic interpretation. In sevoflurane‐related animal models, key markers of ferroptosis—such as GPX4 inactivation, Fe^2+^ accumulation, and increased lipid peroxidation products—typically emerge within hours to a few days following anesthesia [10, 46, 62]. These acute changes primarily reflect the “immediate molecular injury” of ferroptosis at the synaptic level. In contrast, the clinical manifestations of POCD—such as inattention, spatial memory impairment, and executive dysfunction—usually become evident several days to weeks after surgery, and in some elderly patients may persist for 1–3 months, far exceeding the acute phase of ferroptotic activity observed experimentally [63]. This temporal discrepancy suggests that ferroptosis is not a direct or immediate causal factor in clinical POCD, but rather acts through a dynamic “acute initiating injury–subsequent cascade amplification” model that contributes to cognitive decline over time: (1) Acute phase (hours to days): Ferroptotic injury primarily induces oxidative disruption of synaptic membranes (e.g., oxidation of polyunsaturated fatty acids and thinning of postsynaptic densities) and mitochondrial dysfunction. During this period, the brain may temporarily compensate via residual synaptic activity and astrocytic release of neurotrophic factors (e.g., BDNF), masking overt behavioral deficits. Consequently, only molecular and structural abnormalities are detectable experimentally, while cognitive performance appears preserved. However, this initial insult reduces synaptic “cognitive reserve capacity,” rendering the brain more vulnerable to subsequent postoperative challenges such as inflammation and metabolic stress. (2) Although acute ferroptotic signaling diminishes over time following anesthesia, the downstream pathological cascades it initiates may persist. Evidence from POCD models indicates sustained microglial inflammatory activation, characterized by increased IL‐1β and TNF‐α expression, which may interact with ferroptosis‐related synaptic injury to further alter the synaptic microenvironment [64]. In addition, mitochondrial dysfunction and disrupted energy metabolism associated with ferroptosis can further compromise neuronal performance, leading to prolonged impairment of synaptic transmission efficiency [46, 52].

Neglecting this temporal discrepancy risks confining the role of ferroptosis to an “immediate injury mechanism,” thereby underestimating its contribution to delayed cognitive impairment. For instance, if cognitive performance is assessed only during the acute ferroptotic phase (24–48 h postoperatively), the brain's compensatory responses may obscure functional deficits, leading to a false conclusion that ferroptosis is unrelated to cognitive dysfunction. Only by extending the experimental observation window to 2–4 weeks post‐surgery—closer to the clinical onset timeline of POCD—can the combined effects of ferroptosis‐initiated injury and subsequent pathological cascades be accurately captured in the cognitive phenotype. Therefore, mechanistic interpretations must clearly recognize that ferroptosis represents a pivotal initiating event linking sevoflurane anesthesia to delayed POCD. Its primary role lies in disrupting synaptic homeostasis and diminishing cognitive reserve through acute injury, thereby predisposing the brain to later inflammatory and metabolic insults that ultimately drive the emergence of cognitive deficits.

These synaptic‐level impairments ultimately converge to manifest as cognitive deficits. In animal models, enhanced ferroptotic activity induced by sevoflurane exposure has shown a strong correlation with impaired performance in the Morris water maze [46]. Moreover, the occurrence of ferroptosis can activate neuroinflammatory responses, driving microglia to release inflammatory mediators such as IL‐1β and TNF‐α, a mechanism that has been demonstrated in models of brain injury [65]. Notably, ferroptosis is closely linked to mitochondrial oxidative stress, which can compromise synaptic membrane integrity and impair synaptic plasticity [6, 66]. Through multiple converging pathways, ferroptosis simultaneously disrupts neuronal activity, synaptic stability, and network integration, positioning itself as a central mechanism bridging molecular toxicity and systems‐level dysfunction [10, 67]. This interconnected network of mechanisms provides a theoretical foundation for future multi‐targeted intervention strategies. By integrating electrophysiological assessments, synaptic morphology, and behavioral phenotyping, a cross‐scale translational framework—linking ferroptosis activation to synaptic injury and cognitive manifestations—can be established. This integrative structure–function–behavior model enables a pathological assessment system that spans from cellular signaling alterations to system‐level behavioral impairments (Figure S6) [41, 46].

### 3.5 Supporting Roles of Multi‐Omics and Imaging Technologies in Elucidating Ferroptosis Mechanisms

Multi‐omics technologies and advanced imaging approaches have served as powerful tools for investigating ferroptosis mechanisms in the nervous system [68]. Single‐cell RNA sequencing (scRNA‐seq) has enabled the dissection of expression profiles of ferroptosis‐related genes such as ACSL4, GPX4, and SLC7A11 within specific cellular populations, particularly within the hippocampal and prefrontal cortical microenvironments of mice [69]. In recent years, studies using single‐cell sequencing in mouse brain tissue following sevoflurane exposure have identified cell type–specific alterations in ferroptosis‐related gene expression, including changes in SLC7A11 [70].

Spatial transcriptomics further allows for the precise localization of ferroptosis activation signals to specific brain regions or cellular structures [71]. For instance, RNA sequencing studies have reported that sevoflurane exposure alters the expression of ferroptosis‐related genes across multiple hippocampal subregions, such as CA1 and the dentate gyrus (DG), and these molecular changes are accompanied by impairments in synaptic function [52, 72]. Sevoflurane exposure also markedly increases lipid peroxidation and disrupts iron metabolism within the brain [73]. Consistent with these findings, lipidomic analyses have shown that ferroptosis is closely linked to PUFA oxidation and disturbances in phospholipid metabolism [74].

In vivo two‐photon imaging and molecular MRI probes have also been employed to dynamically monitor the spatial distribution and temporal progression of ferroptosis‐associated molecules. When integrated with transcriptomic data, these modalities help construct a multimodal functional network linking ferroptosis to synaptic injury [75, 76]. In summary, multi‐omics and imaging technologies have not only enhanced the resolution of mechanistic investigations into ferroptosis but also facilitated its translational progression from basic pathology to precise therapeutic targeting.

### 3.6 Temporal Window Mechanisms for Ferroptosis‐Targeted Intervention

Recent studies suggest that anesthesia‐related ferroptosis is not an instantaneous event but rather a dynamically evolving process. Across different perioperative stages, changes such as reduced GPX4 expression, increased ACSL4 levels, and accumulation of mitochondrial ROS have been observed, indicating that ferroptotic activity persists and progressively intensifies after anesthesia [77, 78].

Most existing intervention studies have focused on immediate or short‐term treatment after surgery, and ferroptosis inhibitors such as Liproxstatin‐1 and Ferrostatin‐1 have demonstrated significant neuroprotective effects in animal models [79, 80]. However, therapeutic efficacy varies substantially depending on the timing of administration, suggesting that a strategy based on “dynamic monitoring and timely intervention” may be more effective than delivering treatment at a fixed time point [77]. To date, systematic time‐series investigations are still lacking, and the temporal dynamics that define the optimal intervention window remain to be elucidated.

### 3.7 Studies Reporting No Association Between Cognitive Impairment and Ferroptosis Markers, and Instances Where Ferroptosis Inhibition Failed to Confer Neuroprotection

Under specific experimental conditions, some studies have not detected an association between cognitive impairment and core ferroptosis markers. Such findings are mainly observed in three settings—non‐sevoflurane anesthesia models, young healthy animals, and particular comorbidity models:

1. *Propofol anesthesia*: In adult rats exposed to a single propofol anesthesia, hippocampal TNF‐α levels increased, and caspase‐3 and c‐fos expression were elevated [81], suggesting that repeated propofol anesthesia can augment central inflammatory mediators and hippocampal neuronal apoptosis. However, these injuries had no evident long‐term effects on neurodevelopment or cognitive function.
2. In sevoflurane anesthesia experiments using healthy mice, short‐term exposure reduces the novel object recognition index; however, current studies have not detected clear changes in canonical ferroptosis markers [82]. Researchers speculate that the highly active antioxidant systems in young animals—such as SOD2 and CAT—may compensate for the mild oxidative stress induced by sevoflurane, thereby preventing substantial activation of the ferroptotic cascade. Under these conditions, cognitive impairment may arise primarily from synaptic dysfunction or mitochondrial metabolic disturbances rather than being directly driven by ferroptosis.
3. In sevoflurane‐anesthetized diabetic mice, significant cognitive deficits have been reported [83], but mechanistic investigations have mainly focused on oxidative stress and the AGEs/RAGE/NF‐κB inflammatory pathway. Direct evidence for ferroptosis involvement is still lacking, suggesting that in the context of metabolic stress, cognitive impairment may be predominantly mediated by non‐ferroptotic mechanisms.

Additionally, iron accumulation has been implicated in the etiology and progression of multiple neurodegenerative diseases (NDDs), and an iron‐dependent, regulated form of cell death—ferroptosis—may be a key contributor. Despite substantial preclinical and clinical evidence supporting a role for ferroptosis in NDDs, therapeutic targeting remains unresolved, including which proteins to target, how to identify clinically relevant biomarkers, and which patient subgroups are most likely to benefit [84].

In summary, these counterexamples do not refute the role of ferroptosis in cognitive impairment but rather highlight its context‐dependent nature: (1) Conditional contribution: The extent to which ferroptosis contributes to cognitive deficits varies according to anesthetic agent, animal age, and underlying disease state. Its influence is more pronounced in sevoflurane‐induced models involving aged or developing animals, whereas in non‐sevoflurane anesthesia, young healthy subjects, or models dominated by apoptosis or severe inflammation, the effects of ferroptosis may be masked or attenuated. (2) Determinants of therapeutic efficacy: The neuroprotective effect of ferroptosis inhibition depends on meeting three critical conditions—intervention timing aligned with the pathological course, drug targets matching core molecular deficits, and absence of interference from stronger pathological pathways. Failure to satisfy these conditions may render ferroptosis inhibition ineffective. These insights underscore that future POCD mechanistic studies should avoid single‐mechanism attribution and instead perform stratified analyses that integrate both experimental context and clinical heterogeneity. Meanwhile, ferroptosis‐targeted interventions should advance toward precision‐based strategies, in which preoperative assessment of ferroptotic activity (e.g., measuring peripheral GPX4 and ACSL4 levels) guides the selection of appropriate inhibitors and optimal intervention windows, thereby enhancing the translational potential of such approaches in clinical settings.

## 4 Ferroptosis‐Targeted Nanodrug Delivery System Design Strategies

### 4.1 Classification of Nanocarriers and Optimization of Delivery Characteristics: A Dual Strategy of Synthetic and Biomimetic Platforms

The key to designing a ferroptosis‐targeted nanodrug delivery system lies in optimizing targeting efficiency, biocompatibility, and delivery stability of the nanomaterials (Table S4) [11, 85]. Current mainstream approaches can be broadly categorized into two types: synthetic nanoplatforms and biomimetic nanoplatforms, each exhibiting distinct advantages in structural tunability and biological behavior [12, 86].

1. Synthetic platforms, including liposomes and polymeric NPs, have been widely used for central nervous system drug delivery due to their high controllability and production scalability [12]. Liposomes, owing to their cell membrane‐like structure and strong lipophilicity, serve as classical systems for brain‐targeted delivery [87]. Lipid‐based nanosystems modified with ligands such as RVG29 have been shown to markedly enhance the ability of Ferrostatin‐1 to penetrate the blood–brain barrier and accumulate within brain tissue, thereby improving its anti‐ferroptotic efficacy [12]. In models of anesthesia‐related cognitive impairment, Liproxstatin‐1 has been reported to ameliorate learning and memory deficits while correcting ferroptosis‐associated alterations, including disturbances in iron metabolism and lipid peroxidation [88].

Polymeric nanoparticles, such as PLGA‐based systems, have been widely applied for central nervous system drug delivery owing to their stability and controlled‐release properties [87]. In ferroptosis‐related brain injury models, several nanocarrier platforms have also been reported to exert neuroprotective effects by modulating pathways such as GPX4 [89]. Liproxstatin‐1 has been shown to improve learning and memory performance in models of anesthesia‐related cognitive dysfunction while reducing ferroptosis‐associated markers [88]. In addition, lipid‐based carriers and polymeric platforms such as liposomes and PLGA nanoparticles exhibit promising brain‐targeting and sustained‐release capabilities, making them suitable for enhancing the stability and cerebral accumulation of small‐molecule ferroptosis inhibitors [12, 87].

2 Biomimetic platforms, including exosomes and biomimetic membrane systems, utilize naturally derived materials to enhance the biological adaptability of delivery systems by reducing immunogenicity and improving targeting specificity [90, 91]. Extracellular vesicles, as membrane‐bound carriers secreted by cells, possess native lipid compositions and surface proteins that enhance their biodistribution and facilitate traversal across multiple biological barriers, thereby improving tissue targeting [90, 91]. Existing studies have shown that sevoflurane can induce ferroptosis in the hippocampus and impair cognitive function, suggesting that interventions targeting ferroptotic pathways may help mitigate perioperative brain injury. These findings provide a theoretical basis for employing exosome‐based delivery systems to transport ferroptosis inhibitors [41].

Biomimetic membrane systems enhance the selective accumulation of nanocarriers at pathological sites by coating them with membranes derived from disease‐associated cells—such as inflamed cerebrovascular endothelium or tumor cells—and leveraging mechanisms like “homotypic adhesion” and receptor‐mediated recognition [92]. In principle, cloaking nanoparticles with neuronal or other brain‐derived cell membranes could promote their homing within neural networks while reducing clearance by monocyte–macrophage systems. Such an approach may provide a biomimetic basis for coordinated modulation of ferroptosis and inflammatory pathways.

In summary, synthetic platforms emphasize structural tunability and delivery timing, whereas biomimetic platforms prioritize targeting specificity and immune evasion. Future research may focus on integrating the structural controllability of synthetic carriers with the biological recognition capabilities of biomimetic systems. For example, combining PEG‐PLGA scaffolds with neuronal membrane coating strategies may enhance target affinity and metabolic stability within brain tissues (Figure S7).

### 4.2 Strategies for Brain Targeting: BBB Penetration Mechanisms and Ligand Modification Techniques

The BBB, composed of tightly joined cerebral microvascular endothelial cells, astrocytic endfeet, and a basement membrane, selectively prevents the entry of macromolecules and hydrophilic compounds into the brain parenchyma, thereby limiting the efficiency of nanodrug delivery to the central nervous system [93]. To achieve effective intracerebral delivery of ferroptosis inhibitors such as Fer‐1 and Liproxstatin‐1, researchers have primarily employed three strategies: ligand modification, enhancement of physical penetration mechanisms, and selection of carriers with inherent BBB‐crossing capabilities [87].

The rabies virus glycoprotein (RVG) peptide, a commonly used brain‐targeting ligand, binds to nicotinic acetylcholine receptors on neuronal membranes to facilitate BBB translocation [94, 95]. Existing studies have shown that PLGA nanoparticles modified with RVG can markedly enhance drug accumulation in brain tissue and improve the efficiency of nose‐to‐brain delivery [96, 97]. Nanocarriers modified with transferrin receptor (TfR) antibodies exhibited substantial BBB penetration via receptor‐mediated transcytosis and achieved widespread distribution within brain parenchyma [98]. Notably, TfR1 also plays a crucial role in the ferroptosis process and serves as a key target for delivering anti‐ferroptotic agents such as Fer‐1 [99].

In addition, recent studies have employed cell‐penetrating peptides (CPPs), such as TAT and Angiopep‐2, to enhance BBB permeability, while incorporating pH‐sensitive domains to control the release of therapeutic agents in the neutral bloodstream versus the acidic intracerebral environment [93, 100]. Several physical penetration techniques, including ultrasound‐assisted delivery and magnetically guided systems, have also been explored to facilitate nanodrug transport. However, due to their high complexity in clinical application, these approaches remain secondary to chemical or biological ligand‐based targeting, which currently serves as the most practical method [101, 102]. Overall, ligand modification strategies have achieved a favorable balance between clinical feasibility and delivery efficiency, making them the most promising approach for BBB penetration to date.

### 4.3 Intelligent Responsive System Design: ROS/pH/Enzyme‐Triggered Release Platforms

In sevoflurane‐induced neural injury, brain tissue exhibits localized accumulation of ROS along with disturbances in iron metabolism [103]. Intelligent responsive nanoplatforms developed in response to these pathological features have emerged as a promising strategy for site‐specific delivery of ferroptosis inhibitors within the brain [46, 88, 104]. Among these, the most prevalent design involves ROS‐responsive materials, such as selenium/sulfur bonds, boronic esters, or redox‐sensitive polymers, which encapsulate the drug and undergo rapid disintegration in high‐ROS microenvironments [104]. This approach effectively prevents premature drug release during systemic circulation while ensuring precise accumulation at sites of ferroptosis activation.

pH‐responsive delivery systems exploit the acidic microenvironment of pathological tissues, where pH‐triggered materials can enhance local drug release efficiency. For example, PEI‐based pH‐responsive nanocarriers exhibit markedly accelerated drug release at pH 6.5–6.8 [104]. Enzyme‐responsive platforms, such as nanocarriers activated by MMP‐9 or Cathepsin B, can further improve site‐specific drug release while minimizing off‐target exposure [104]. In addition, several nanoplatforms have achieved “temporal matching release” within ferroptosis‐active regions; for instance, ROS‐responsive delivery systems can release Fer‐1 in a staged manner in response to elevated oxidative stress at diseased sites, thereby enhancing the spatiotemporal precision of ferroptosis inhibition (Figure S8) [105].

In summary, intelligent responsive systems, by aligning with disease‐specific pathological cues, markedly improve the spatial and temporal precision of drug release and enhance tissue specificity, thereby reducing systemic toxicity and off‐target burden [79, 105]. Multiple studies have validated the application of such systems for ferroptosis inhibitor delivery in POCD animal models, and several platforms have advanced into preclinical evaluation stages [106].

### 4.4 Target‐Carrier Matching Strategy and Design Pathways

The diversity of ferroptosis‐related targets necessitates highly specific nanocarrier designs [107]. Current ferroptosis intervention strategies primarily focus on three molecular targets: GPX4 activation, ACSL4 inhibition, and SLC7A11 agonism. Each target imposes distinct requirements regarding the physicochemical properties, release kinetics, and intracellular localization of the drug, thereby demanding a customized matching of carrier systems [67]. Existing studies suggest that different ferroptosis targets may require distinct delivery strategies. Interventions aimed at SLC7A11 often depend on enhancing drug stability; thus, PEGylation or stimulus‐responsive nanocarriers are commonly employed to improve encapsulation efficiency [107, 108].

Regarding structural construction, the most established approach is a “core‐shell‐functionalization” tri‐layer design. This architecture integrates a polymeric core for controlled release, a lipid shell that mimics the cellular membrane environment, and a functional outer layer that enables brain targeting and stimulus responsiveness [102]. Some studies have utilized click chemistry or ester bond linkages to covalently attach targeting ligands to the carrier surface, thereby enhancing system stability and controllability [109]. Moreover, the emerging class of “self‐assembling peptide NPs” has been increasingly applied in ferroptosis‐targeted delivery systems. These platforms offer programmable sequences and tunable functionalities, making them particularly suitable for constructing multi‐target regulatory platforms [110].

In summary, the essence of target‐carrier matching lies in reverse‐engineering the nanocarrier design based on pathological features to enhance drug bioavailability, prolong therapeutic duration, and improve brain‐targeting precision. This strategy provides a technical foundation for precise modulation of ferroptosis mechanisms.

### 4.5 Challenges in Delivering Ferroptosis Inhibitors: Stability, Controlled Release, and Biocompatibility

Although ferroptosis‐targeted drug delivery systems offer significant theoretical advantages, their practical application remains hindered by several technical and biological challenges. The first major issue is the in vivo stability of drug‐loaded systems. Most ferroptosis inhibitors are lipophilic small molecules, which tend to bind plasma proteins or undergo esterase‐mediated hydrolysis, resulting in premature drug release [111, 112]. Even when encapsulated within nanocarriers, inadequate loading efficiency or insufficient surface modification may still lead to early drug leakage in plasma, causing reduced efficacy and nonspecific tissue toxicity, with a persistent risk of drug loss during systemic circulation [113].

Second, controlled release performance is critical for maintaining therapeutic efficacy and ensuring safety. Since ferroptosis‐related processes can persist from several hours to days after anesthesia, drug release must be both delayed in onset and sustained over time [114]. Furthermore, and more critically, poor long‐term biocompatibility remains a central bottleneck that has historically hindered the clinical translation of neuro‐nanomedicines. Multiple preclinical programs have been terminated due to inadequate evaluation of chronic safety, underscoring the need for rigorous long‐term assessments. Representative examples include: (1) Chronic toxicity risk of quantum dots: When quantum dots (QDs) persist intracellularly or accumulate over time in vivo, their protective coatings may degrade, generating “naked” QDs. These exposed particles induce damage to the plasma membrane, mitochondria, and nucleus, ultimately leading to cell death [115]. Thus, although QDs—owing to their small size and fluorescent imaging capability—hold promise for diagnosis, monitoring, and therapy of neurological disorders, concerns about their toxicity remain unresolved [116]. (2) Long‐term neurotoxicity of cationic polymers: Polyethylenimine (PEI)‐modified magnetic PLGA nanoparticles have been shown to enhance drug delivery efficiency [117], and novel PEI formulations exhibit substantial potential to overcome current barriers in PEI‐mediated gene delivery during clinical translation, particularly with respect to target specificity, efficacy, and long‐term stability [116].

In conclusion, future research should pursue multi‐level optimization strategies—including material design, drug selection, delivery route engineering, and immune modulation—to achieve safe and efficient clinical translation of ferroptosis inhibitor‐based nanodelivery systems [118].

## 5 Cross‐Model Evidence for Nanotechnology‐Mediated Ferroptosis Modulation and Its Potential Application in POCD

As a key pathological process underlying sevoflurane‐induced synaptic injury and cognitive dysfunction, ferroptosis has emerged as an important neuroprotective target in the perioperative period [8, 10]. However, the clinical utility of conventional small‐molecule ferroptosis inhibitors remains limited due to poor penetration of the blood–brain barrier (BBB), short in vivo half‐life, and insufficient regional targeting within the brain [93, 119]. Nanodelivery platforms provide a promising technological approach to overcoming these barriers, enabling “precision ferroptosis modulation” through intelligent responsiveness, targeted recognition, and controlled release [93, 120]. The following section synthesizes the potential value and research directions of nanotechnology‐based systems for ferroptosis intervention from three complementary perspectives: underlying technological principles, mechanisms of action, and cross‐model experimental evidence.

### 5.1 Design Principles and Intervention Pathways of Nanotechnology‐Based Ferroptosis Modulation

Nanodelivery systems offer distinct advantages in crossing the blood–brain barrier (BBB) and achieving targeted drug delivery. Studies have shown that nanoplatforms designed with pH‐responsive, receptor‐mediated transport, or biomimetic membrane strategies can bind to specific receptors on the BBB surface, thereby markedly enhancing drug accumulation within the central nervous system [120]. Under acidic microenvironments or oxidative stress conditions, these carriers can trigger on‐demand drug release, reducing excessive systemic exposure and peripheral toxicity, and thus providing an effective delivery platform for ferroptosis inhibitors such as Ferrostatin‐1 and Liproxstatin‐1 [121].

Moreover, intelligent stimulus‐responsive nanocarriers not only facilitate targeted delivery of ferroptosis inhibitors but also offer controllable release kinetics and responsiveness to local microenvironmental changes. These features establish a technical foundation for developing next‐generation systems capable of monitoring the dynamic progression of ferroptosis [121, 122]. It should be noted that most mechanistic validations of these nanoplatforms in ferroptosis modulation originate from tumor, ischemic brain injury, or neuroinflammation models. Their roles in POCD remain extrapolative at this stage; however, these findings provide transferable design principles and optimization strategies for future POCD‐specific investigations.

### 5.2 Cross‐Model Experimental Evidence for Nanotechnology‐Based Ferroptosis Intervention

Under conventional (non‐nanocarrier) drug administration, numerous perioperative neural injury models have demonstrated that ferroptosis inhibitors significantly ameliorate synaptic damage and behavioral deficits, providing a solid pharmacological foundation for developing nanodelivery strategies for these agents [79, 80, 88].

In sevoflurane‐induced POCD models, both Ferrostatin‐1 and Liproxstatin‐1 markedly reduce oxidative stress, restore GPX4 activity, alleviate hippocampal synaptic structural damage, and improve performance in the Morris water maze [79]. Additional evidence shows that suppressing iron deposition and preventing GPX4 inactivation not only improves short‐term cognitive function but also reverses damage to mitochondrial cristae, indicating that ferroptosis inhibition may confer long‐term synaptic repair potential [88].

Moreover, a “preoperative priming + postoperative maintenance” regimen has been shown to produce superior cognitive protection compared with single‐time‐point intervention in traumatic brain injury models [80]. This finding highlights the temporal dependency of ferroptosis modulation and suggests the existence of a therapeutic “reversibility window,” providing a theoretical basis for time‐sequenced drug delivery using nanoplatforms.

Although no animal studies have yet directly applied nanodelivered ferroptosis inhibitors in POCD models, related research in other neuropathological contexts has established a clear mechanistic chain—nanodelivery → ferroptosis regulation → neurological improvement. For example, pH‐responsive and receptor‐mediated nanocarriers significantly enhance the accumulation of ferroptosis inhibitors within brain parenchyma and, in ischemic brain injury models, reduce lipid peroxidation, preserve mitochondrial ultrastructure, and restore synchronized neuronal network activity [120]. In studies involving neuroinflammation and tumor‐associated ferroptosis, nanodelivery strategies have been shown to modulate oxidative stress, stabilize iron homeostasis, and increase the effective drug concentration at target sites, indicating that “nanoplatforms + ferroptosis targets” possess cross‐disease applicability [123, 124, 125].

Taken together, substantial cross‐model evidence supports the notion that nanodelivery systems can enhance the neuroprotective effects of ferroptosis inhibition. Although this evidence chain has not yet been validated directly in POCD models, it provides a transferable experimental basis for designing POCD‐specific nanointervention strategies.

### 5.3 Multidimensional Evaluation of Nanotechnology‐Based Ferroptosis Modulation: Current Evidence and Consistency Challenges

Current research on ferroptosis‐targeted nanodelivery systems has shifted from evaluating single materials or isolated endpoints to adopting multidimensional and integrated assessment strategies. At the structural and material level, different nanocarrier platforms—such as liposomes, PLGA nanoparticles, and exosomes—exhibit substantial variability in drug‐loading capacity, brain accumulation efficiency, biodegradation rates, and immunogenicity. Some studies suggest that exosome‐based carriers offer potential advantages in neural affinity and BBB penetration; however, issues related to donor heterogeneity, purification complexity, and large‐scale production continue to impede their clinical translation [126, 127].

At the molecular and mechanistic level, ferroptosis‐related targets such as GPX4, ACSL4, and SLC7A11 play distinct roles during pathological progression, leading to considerable differences in optimal intervention strategies and drug types. Review studies further highlight that GPX4 is central to lipid peroxidation defense, ACSL4 determines fatty acid activation and membrane susceptibility, and the system Xc^−^ pathway regulates glutathione replenishment [99]. These insights indicate that matching specific targets with appropriate nanomaterials should serve as a primary step in nanoplatform design.

In terms of functional effects and systematic validation, a recent trend involves integrating multi‐dimensional evaluation systems. Studies combining high‐throughput omics with multi‐parametric behavioral analysis have established a “target modulation—molecular effect—functional improvement” tri‐layer framework [128, 129], reducing reliance on single molecular readouts and improving comparability across different intervention approaches. In this work, we propose a conceptual three‐dimensional evaluation framework encompassing synaptic morphology, molecular mechanisms, and behavioral performance (Figure S9), which may inform individualized strategies for future research on POCD.

Collectively, multidimensional evaluation systems represent a critical foundation for standardizing nanotechnology‐based ferroptosis interventions, improving cross‐study comparability, and enhancing mechanistic interpretation—particularly in light of the substantial heterogeneity across material platforms, molecular targets, and functional outcomes.

### 5.4 Time Window and Personalized Strategies for Ferroptosis Intervention: Research Priorities From a Dynamic Mechanistic Perspective

Current evidence indicates that ferroptosis exhibits strong temporal dependency, involving a sequence of events including iron accumulation, lipid peroxidation, and disruption of antioxidant defenses. Animal studies have shown that, following sevoflurane anesthesia, characteristic ferroptosis markers—such as increased ACSL4 expression, decreased GPX4 levels, and elevated ROS—can be detected, suggesting the involvement of ferroptosis in perioperative neural injury [8]. However, the temporal evolution of these molecular signals remains poorly defined, and a systematic characterization of the “optimal therapeutic window” is still lacking [77].

With respect to intervention strategies, most POCD studies have focused on short‐term, postoperative inhibition of ferroptosis. Treatments using Ferrostatin‐1 (Fer‐1) or Liproxstatin‐1 have been shown to reduce neuroinflammation and improve behavioral outcomes [79]. Other reports indicate that targeting GPX4 inactivation and iron deposition can enhance long‐term learning and memory performance and even reverse mitochondrial structural damage [88]. More recent studies have begun exploring a “preoperative priming plus postoperative maintenance” paradigm, aiming to prolong exposure to ferroptosis inhibitors for sustained neuroprotection. For instance, in traumatic brain injury models, Fer‐1 administered both before and after injury yields more robust cognitive improvements than single‐time‐point treatment [80].

Despite encouraging findings, substantial heterogeneity remains across studies in dosing schedules, timing, and treatment duration, with no unified standard currently available. Review articles emphasize that the temporal progression of ferroptosis is closely shaped by surgical stress, anesthesia, and ischemia–reperfusion events, highlighting the need for a framework based on dynamic monitoring and timely intervention [77, 78].

Future research may prioritize two directions. First, constructing a temporal atlas of ferroptosis‐related biomarkers and aligning these trajectories with the drug‐release profiles of nanodelivery systems may allow precise matching of intervention windows with pharmacodynamic activity. Second, incorporating ROS‐responsive imaging probes, fMRI, multimodal behavioral assessments, and machine learning models may facilitate identification of intraoperative peaks in ferroptotic activity and support preoperative risk stratification and postoperative follow‐up.

Overall, ferroptosis intervention strategies should transition from “fixed treatment time points” to dynamic, time‐sequence modulation and personalized precision approaches. Such a shift may substantially improve the feasibility and translational potential of nanotechnology‐based ferroptosis modulation in clinical POCD management.

### 5.5 Integrative Evidence and Mechanistic Inference: Potential Implications of Nanotechnology‐Based Ferroptosis Modulation in POCD

Drawing on the cross‐model evidence and mechanistic findings summarized above, this section presents a conceptual inference regarding how nanodelivery of ferroptosis inhibitors might function in the context of POCD. Importantly, the discussion below is prospective and hypothesis‐driven, rather than a summary of existing experimental outcomes. Its purpose is to outline a theoretical framework and testable hypotheses to guide future research. Overall, nanotechnology‐based interventions may influence ferroptosis through three interconnected dimensions: enhanced delivery efficiency, coordinated modulation of multiple targets, and optimization of therapeutic timing.

At the level of drug delivery, conventional ferroptosis inhibitors exhibit limited exposure, short duration of action, and insufficient regional targeting in POCD models, resulting in poor spatial–temporal alignment between drug activity and pathological ferroptosis dynamics. Nanocarriers capable of crossing the BBB—through receptor‐mediated transport, ligand modification, or controlled‐release strategies—have the potential to increase drug accumulation in cognitively relevant regions such as the hippocampus and prefrontal cortex, thereby establishing the spatial foundation necessary for ferroptosis modulation.

At the target‐regulation level, ferroptosis associated with POCD involves multiple pathological components, including GPX4 inactivation, enhanced lipid peroxidation, upregulation of ACSL4, and disruption of iron homeostasis. Nanodelivery platforms allow integration of synergistic strategies—such as antioxidant delivery, iron‐metabolism rebalancing, and mitochondrial protection—thereby extending intervention from a single molecular target to a coordinated, multi‐pathway regulatory approach. This may provide a more stable biological basis for synaptic preservation and restoration of neural network function.

At the temporal level, ferroptotic activity in POCD appears to follow a dynamic, stage‐dependent pattern, with acute surges followed by subacute persistence. The sustained‐release properties of nanocarriers may better align therapeutic exposure with these temporal rhythms, enabling “cross‐phase continuous blockade” of ferroptosis. Such alignment may enhance the durability of synaptic repair and behavioral recovery.

Taken together, while direct experimental evidence for nanodelivered ferroptosis inhibitors in POCD is still lacking, cross‐model findings suggest that nanotechnology offers advantages in achieving more stable brain exposure, more precise target modulation, and more flexible temporal control. Future studies should develop systematic experimental designs using aged and comorbidity‐associated POCD models, combined with multimodal evaluation approaches—including behavioral assays, electrophysiology, multi‐omics, and imaging—to determine the true mechanisms and clinical feasibility of nanotechnology‐based ferroptosis modulation in POCD.

## 6 Clinical Translation Pathways and Strategies for Individualized Intervention

### 6.1 Clinical Assessment Criteria for POCD and Identification of Susceptible Populations

Currently, there is no universally accepted gold standard for diagnosing POCD, and clinical evaluation typically relies on the combined use of multidomain neuropsychological assessment tools [130, 131]. The International Study of Postoperative Cognitive Dysfunction (ISPOCD) group introduced a classical multidomain neuropsychological test battery designed to assess changes in executive function, memory, attention, and information‐processing speed. It remains one of the most widely used standardized tools in POCD research [132]. In addition, recent guidelines recommend incorporating longitudinal assessments at multiple pre‐ and postoperative time points, as well as evaluations of everyday functional abilities, to enhance the ecological validity of cognitive outcome measurements [133].

Although peripheral blood miRNAs such as miR‐21 and miR‐29a have demonstrated promising predictive and stratification value in cancer surgery patients [134], miRNA‐based predictive models for POCD remain highly limited, with no definitive evidence yet supporting their clinical application. In related fields, proteomic and metabolomic profiling of exosomes has shown potential for monitoring neuroinflammation and blood–brain barrier injury in perioperative delirium (POD) research [135]. However, for POCD, reliable and reproducible preoperative biomarkers are still lacking, and exosome‐based liquid biopsy platforms require further validation before they can be considered for clinical use.

In the future, the development of individualized intervention strategies will increasingly rely on predictive platforms of this kind, providing a basis for preclinical selection of candidates for ferroptosis‐targeted therapies.

### 6.2 Interindividual Variability of Ferroptosis Targets and the Potential for Precision Intervention

Existing studies have shown that the expression of key ferroptosis‐related molecules—such as GPX4, SLC7A11, and ACSL4—undergoes marked alterations across different pathological conditions and directly influences the susceptibility of neural tissue to oxidative stress and lipid peroxidation injury [136, 137]. Moreover, various pathological states can significantly modify the activity of iron metabolism and oxidative stress pathways. For example, in populations with conditions such as male infertility or sarcopenia, aberrant expression patterns of ACSL4, SLC7A11, and GPX4 have been documented, suggesting that comorbidity itself may reshape ferroptosis vulnerability [138, 139].

Precision intervention strategies rely on tailoring drug combinations and dosage regimens based on the individual's specific “ferroptosis activity landscape.” For instance, in individuals with markedly reduced GPX4 expression, lipid‐targeted antioxidants such as Liproxstatin‐1 should be prioritized. In contrast, for those exhibiting upregulation of ACSL4, therapeutic strategies should focus on preserving membrane lipid integrity. Transcriptomic and proteomic analyses of clinical samples have provided preliminary evidence supporting this subtype‐based approach [140]. In the future, integrating magnetic resonance imaging radiomics with peripheral multi‐omics signals may enable the development of a triadic precision intervention framework encompassing prediction, monitoring, and treatment.

### 6.3 Technical and Regulatory Challenges in the Clinical Application of Nanodrug Delivery Systems

Although nanodrug delivery systems hold significant promise for the treatment of neurological disorders, their clinical translation remains hindered by several fundamental challenges. The first major issue involves immunogenicity and toxicity. Certain conventional synthetic polymers—such as cationic polymers and biodegradable polyesters—may trigger immune recognition or accumulate within tissues during in vivo use. Ongoing research is therefore exploring the adoption of natural exosomes and biomimetic membrane strategies to minimize immunogenic responses [141, 142].

The second challenge lies in the unclear pathways of biodegradation and metabolism. Limited data from animal models exist regarding how most nanocarriers are degraded, metabolized, and cleared following central nervous system administration [143]. International regulatory agencies, including the U.S. Food and Drug Administration (FDA) and the European Medicines Agency (EMA), require comprehensive evaluations of nanomedicines encompassing long‐term toxicity, tissue distribution, and metabolite‐associated toxicity, and they emphasize the need to establish quality control systems that operate independently from those used for conventional drugs [119]. This is particularly critical for nanocarriers delivering ferroptosis inhibitors, which closely interact with mitochondria and iron metabolism, necessitating heightened vigilance toward potential cellular stress‐related side effects [93].

The third major obstacle concerns production consistency and batch‐to‐batch reproducibility. The core obstacle to manufacturing consistency lies in the instability of process scaling from laboratory‐scale synthesis to industrial production—a challenge particularly pronounced in neural‐targeted nanocarrier systems. Historically, multiple liposomal therapeutics have stalled in clinical translation due to such scale‐up inconsistencies. A key issue involves size and encapsulation efficiency variability during scale‐up. For instance, laboratory‐grade liposomes prepared via a syringe‐pump method (30 mL batch) exhibit a uniform particle size of 82–95 nm and an encapsulation efficiency of 86.4% for lipophilic drugs. However, when scaled up to pilot‐scale production (750 mL batches), changes in stirring dynamics and solvent diffusion rates lead to broadened particle‐size distributions (100–150 nm), an increase in the polydispersity index (PdI) from 0.12 to 0.25, and wider fluctuations in encapsulation efficiency (65%–82%) [144]. Existing studies have shown that nanoparticle size strongly influences BBB penetration efficiency, with smaller particles (20–70 nm) exhibiting markedly higher brain accumulation than larger counterparts [145]. For example, insulin‐targeted gold nanoparticles (INS‐GNPs) in Balb/C mice demonstrated a clear size‐dependent BBB permeability: 20 nm INS‐GNPs exhibited significantly higher brain accumulation 2 h post‐injection compared with 50 and 70 nm particles [145]. While some biomimetic or covalently modified systems demonstrate functional efficacy at the laboratory scale, they often lack standardized processes for large‐scale manufacturing, severely restricting their clinical development. In response, recent efforts have aimed to establish standardized platforms for nanodrug quality control, emphasizing quantifiable and controllable parameters such as particle size, zeta potential, encapsulation efficiency, and release kinetics [146, 147]. The development of Good Manufacturing Practice (GMP) protocols tailored for neurological nanomedicine remains a critical technical milestone for advancing clinical translation.

The issue of batch‐to‐batch reproducibility, which is closely tied to manufacturing consistency, represents another historical stumbling block in the translation of neuro‐nanomedicines. This challenge is particularly evident in multi‐step modified or biomimetic nanocarrier systems, where even subtle deviations in synthesis or source materials can lead to substantial functional variability. Batch variability in exosome‐based carriers: Exosomes derived from neural stem cells often show significant batch‐to‐batch differences in brain‐targeted drug delivery efficiency, primarily due to variations in donor cell culture conditions—including passage number and medium composition [148]. According to the International Society for Extracellular Vesicles (ISEV, 2023), 60% of exosome‐based neurodelivery studies failed to meet the MISEV reproducibility standards, resulting in irreproducible key experimental outcomes [149].

It is important to note that in complex nanocarrier systems, slight variations in grafting efficiency, impurity levels, and reaction conditions during multistep fabrication can substantially alter drug‐release kinetics [150]. This indicates that the performance of multicomponent and functionalized nanoplatforms is highly dependent on the stability and controllability of their manufacturing processes, necessitating strict consistency during scale‐up production.

In addition, variability in excipient quality may also lead to batch failure. For example, changes in impurity levels of certain surfactant excipients have been shown to affect the stability and effective drug loading of nanomedicines [149]. These findings underscore the extreme sensitivity of neuro‐nanomedicines to excipient purity and highlight the need to establish standardized excipient specifications and unified quality‐control guidelines to ensure batch‐to‐batch reproducibility.

### 6.4 Advances in Ferroptosis‐Based Interventions in Clinically Relevant Preclinical Models and the Prospects of Combined Strategies

Although most ferroptosis‐targeted nanointervention strategies remain at the stage of animal model validation, some approaches have begun advancing toward clinically relevant models. In recent years, iPSC‐derived brain organoids and human neuronal models have been increasingly employed to investigate the role of ferroptosis in neurological disorders and to evaluate the potential protective effects of ferroptosis inhibitors [151, 152]. Compared to conventional murine models, such systems more accurately reflect human‐specific features of ferroptosis in the brain.

Based on the regulatory frameworks of the U.S. FDA and EMA for CNS therapeutics and historical translational data, the clinical translation of ferroptosis‐targeted interventions (including nanomedicine delivery systems) can be delineated into five core stages over a 10–15 year timeline. The milestones and validation requirements for each stage are summarized in Table S5:

1. Preclinical validation (2–3 years): establishing the foundation for regulatory submission. This stage must deliver human‐relevant efficacy evidence, long‐term safety data, and mechanism confirmation as prerequisites for submitting an Investigational New Drug (IND) application. Key validation requirements include: (i) Use iPSC‐derived brain organoids and/or humanized immune mouse models to demonstrate that ferroptosis inhibition repairs human‐relevant synaptic injury (at least two models for cross‐validation [153]); (ii) Conduct ≥ 6‐month nonhuman primate (e.g., cynomolgus macaque) toxicity studies, with focused monitoring of nanocarrier accumulation in cognition‐related brain regions (e.g., hippocampus, prefrontal cortex), in accordance with the FDA guidance on nonclinical safety evaluation for CNS drugs; (iii) Establish PK/PD relationships linking intracerebral drug concentrations to cognitive improvement, thereby defining exposure–response relationships.
2. IND submission and Phase I (1–2 years): safety‐first evaluation. Following completion of preclinical studies, an IND is submitted to the FDA/EMA; upon approval, Phase I trials commence (typically enrolling 20–100 healthy volunteers or individuals at elevated risk for mild POCD). Regulatory focus: (i) Assess the safety of single and multiple dosing (e.g., neuroinflammation, hepatic/renal dysfunction), with particular attention to whether the nanocarrier perturbs brain iron homeostasis (monitor serum ferritin and CSF iron [154, 155]); (ii) Characterize pharmacokinetics, including BBB penetration and intracerebral half‐life, often quantified with PET imaging to map brain distribution [156]. Time bottleneck: If unexpected neurotoxicity occurs (e.g., headache, transient cognitive decline), the program typically reverts to preclinical carrier optimization, adding ~6–12 months to timelines.
3. Phase II (2–3 years): preliminary efficacy and dose‐finding. This stage enrolls 100–300 patients with confirmed POCD to establish initial efficacy and determine the optimal dose. Key design elements: (i) Randomized, double‐blind, placebo‐controlled trials using 3‐month postoperative cognitive change (e.g., MoCA increase ≥ 2 points) as the primary endpoint; (ii) Subgroup analyses (e.g., older adults/children, patients with diabetes) to inform Phase III population selection; (iii) Extended safety follow‐up (≥ 6 months) to detect delayed intracerebral accumulation. Failure risk: According to FDA experience, ~40% of CNS drugs terminate in Phase II for insufficient efficacy; for example, one ferroptosis inhibitor halted in 2022 when the MoCA gain (1.2 points) fell short of the prespecified 1.8‐point threshold.
4. Phase III (3–4 years): large, multicenter confirmatory trials. This is the most time‐consuming and costly stage of clinical translation, typically enrolling 1000–3000 patients across multiple countries/regions to confirm efficacy and safety. Regulatory priorities: (i) Demonstrate the causal chain of “ferroptosis inhibition → synaptic protection → cognitive improvement,” integrating biomarkers (e.g., CSF GPX4 activity, synapse‐related protein levels) with neuroimaging (e.g., fMRI functional connectivity) evidence; (ii) Evaluate drug–drug interactions with concomitant medications (e.g., synergy or antagonism with postoperative analgesics); (iii) Submit a complete manufacturing process validation dossier, including batch‐to‐batch reproducibility data for the nanocarrier system. Time burden: Multicenter coordination and patient recruitment (particularly the challenges of POCD follow‐up) often prolong timelines by an additional ~6–9 months beyond initial projections.
5. Marketing authorization and Phase IV surveillance (1–2 years): comprehensive risk management. Upon successful completion of Phase III, sponsors submit a New Drug Application (NDA) or Biologics License Application (BLA). After approval, products enter Phase IV (post‐marketing) surveillance. Approval focus: Regulators (FDA/EMA) scrutinize the long‐term benefit–risk profile, including whether the 10‐year accumulation risk of the nanocarrier remains lower than the cognitive benefit achieved. Orphan drug designation (e.g., for pediatric POCD) may qualify for priority review, but pediatric PK data are still required. Phase IV requirements: Monitor ≥ 10,000 patients for long‐term safety over 2–5 years, with special attention to potential induction of neurodegenerative pathology (e.g., aberrant tau phosphorylation). Detection of serious adverse events may trigger market withdrawal (Table S5).

On the other hand, increasing attention has been directed toward the synergistic interaction between ferroptosis‐targeted interventions and the regulation of inflammatory pathways. For example, existing studies suggest that ferroptosis intersects with immune‐related mechanisms such as NLRP3 inflammasome activation and pyroptosis, indicating bidirectional regulatory relationships [152, 157]. In addition, combining intelligent stimulus‐responsive nanoplatforms with exogenous neuromodulatory approaches—such as near‐infrared photostimulation—has been shown to enhance synaptic plasticity and promote functional recovery [158], suggesting that nanotechnology offers promising compatibility with neural modulation–based therapeutic strategies. Such multi‐pathway, network‐level interventions are well aligned with the complex pathophysiological mechanisms underlying POCD, and they represent a forward‐looking direction in the pursuit of precision therapeutics. With the rapid progress of AI‐assisted drug screening, molecular imaging‐guided delivery monitoring, and integration of real‐world clinical datasets, ferroptosis‐targeted strategies are expected to continue evolving across preclinical and clinical stages, enabling a critical transition from mechanistic targeting to individualized therapeutic efficacy.

Although ferroptosis‐targeted interventions have demonstrated promising neuroprotective potential in preclinical models, it is essential to recognize the realistic temporal scale of translational development. For novel neuroprotective strategies, including nanomedicine‐based delivery systems, the journey from mechanistic validation to clinical application typically spans 10–15 years—far exceeding the expectations often set in basic research for “rapid translation.” According to statistics from journals such as Stroke and Neurotherapeutics, the average translational timeframe for neuroprotective drugs targeting central nervous system (CNS) disorders is approximately 12.3 years. For nanocarrier‐based systems, this process is typically extended by an additional 1–2 years, due to the need for rigorous evaluation of BBB penetration safety, long‐term bioaccumulation risks, and manufacturing reproducibility. Even for ferroptosis inhibitors showing the fastest progress—such as Fer‐1 derivatives—their nanodelivery formulations remain in the early preclinical validation phase. Consequently, their clinical application is expected to require at least another decade, contingent on successfully passing multiple regulatory milestones and multiphase validation studies to ensure safety, efficacy, and scalability.

## 7 Conclusion and Future Directions

### 7.1 Establishment of an Integrated Framework Linking Ferroptosis, Synaptic Injury, and Intelligent Nanointervention

POCD, a major manifestation of perioperative neurological injury, has seen a paradigmatic shift in its underlying mechanisms, from traditional theories centered on neuronal apoptosis to emerging evidence highlighting synaptic plasticity impairment. As the fundamental structural unit regulating cognitive behavior, synapses have been identified as the most prominently affected targets of sevoflurane anesthesia in recent years. Ferroptosis, a form of programmed cell death driven by lipid peroxidation, has shown strong associations with synaptic membrane lipid oxidation, decreased expression of synaptic proteins, and disruption of synaptic networks, and has emerged as a critical mechanistic explanation for the neurotoxicity observed in POCD.

This review represents the comprehensive integration of ferroptosis signaling and synaptic injury mechanisms, proposing ferroptosis as a central pathological nexus in POCD. We construct a triadic intervention framework that links impaired synaptic plasticity, ferroptosis activation, and precision nanodrug delivery, forming a coherent system that spans molecular mechanisms, functional imaging, and intelligent nanointervention strategies. Notably, existing literature lacks a systematic review that simultaneously incorporates ferroptosis, synaptic‐level pathology, and intelligent responsive nanoplatforms for targeted intervention. The present study distinguishes itself by offering a deeply integrative and multi‐dimensional perspective that bridges mechanistic insights with translational nanotechnology.

### 7.2 Technological Potential and Expanding Applications of Nanodrug Delivery Systems in Neuroprotection

The development of nanodelivery systems has not only helped overcome the challenge of poor blood–brain barrier penetration associated with small‐molecule ferroptosis inhibitors but has also provided new platforms for targeted release and pharmacological visualization. In particular, emerging delivery strategies—including ROS‐ and pH‐responsive systems, cell membrane–mimicking nanocarriers, and exosome‐based transport—exhibit improved brain accumulation and controllable drug‐release profiles in central nervous system applications. These technologies offer a robust foundation for delivering neuroprotective agents such as ferroptosis inhibitors [120].

Moreover, intelligent nanocarriers are increasingly expanding their functional scope, transitioning from “passive delivery” systems to “multi‐modal intervention” platforms. Strategies integrating the co‐delivery of neurotrophic factors, stimuli‐responsive imaging probes, and self‐destructive carrier designs are actively reshaping POCD interventions, shifting the paradigm from single‐target pharmacologic modulation to network‐level regulation and real‐time feedback monitoring. These systems are expected to serve as critical bridges between fundamental mechanistic research and clinical translation in the near future.

### 7.3 Future Research Directions

To further enhance the precision, translational potential, and generalizability of ferroptosis‐targeted interventions, future investigations may advance along four key directions:

1. Mechanistic Expansion: Emphasis should be placed on exploring the crosstalk between ferroptosis and other forms of programmed cell death, such as pyroptosis, cuproptosis, and necroptosis. Existing studies have demonstrated that ferroptosis intersects with pyroptotic and inflammatory pathways through mitochondrial stress, calcium overload, and lipid signaling. It is advisable to construct an integrative interaction map of cell death pathways and develop a “ferroptosis‐pyroptosis‐inflammation” regulatory model to elucidate the multifaceted pathological mechanisms of POCD and strengthen the multilayered targeting of therapeutic strategies [159].
2. Material Optimization: Artificial intelligence (AI) and omics technologies should be combined to facilitate the screening of personalized nanointervention systems. As omics platforms continue to evolve, it has become feasible to construct individualized ferroptosis activity profiles for high‐risk POCD populations. The integration of AI algorithms is recommended to model the relationships among patient‐specific omics data, carrier configurations, and pharmacodynamic outcomes, thereby enabling a dual‐track platform of virtual prediction and experimental validation to improve nanomaterial‐target compatibility [160].
3. Accelerated Translation: Efforts should be directed toward establishing humanized POCD models and dynamic cognitive imaging evaluation systems. Traditional rodent models face limitations in pathological comparability. It is therefore recommended to adopt iPSC‐derived cerebral organoids or humanized immune‐humanized mouse models to replicate sevoflurane‐induced synaptic network damage. Concurrently, the use of multimodal imaging technologies—such as fMRI, two‐photon microscopy, and magnetic resonance spectroscopy—should be promoted to monitor cognitive changes, forming an integrated evaluation chain from “intervention to imaging to cognition.” Ethical review and regulatory frameworks must be developed in parallel to support this system [161].
4. Clinical Integration: A dynamic intraoperative‐postoperative ferroptosis monitoring and stratified intervention system should be established. By integrating single‐cell transcriptomics and spatial omics with intraoperative electroencephalography and postoperative MRI‐based radiomics, it becomes possible to achieve preoperative risk prediction, intraoperative targeted delivery, and postoperative efficacy tracking in POCD patients. It is recommended that a monitoring system for ferroptosis‐related biomarkers be established, with particular attention to molecular indicators such as GPX4 activity and MEF2C expression for perioperative risk assessment [162].

## Author Contributions

B.Z. conceived and designed the study. B.Z., C.W., and M.Z. performed the experiments. B.Z. analyzed the data. B.Z., C.W., and M.Z. wrote the manuscript. All authors reviewed and approved the final version of the manuscript.

## Funding

This study was supported by the Liaoning Provincial Joint Fund Project (No. 2023‐MSLH‐126).

## Ethics Statement

The authors have nothing to report.

## Conflicts of Interest

The authors declare no conflicts of interest.

## Supporting information


**Figure S1:** Mechanistic illustration of synaptic plasticity vulnerability during developmental and aging stages.

**Figure S2:** Mechanistic illustration of regional brain dysfunction induced by sevoflurane‐related POCD.

**Figure S3:** Canonical ferroptosis pathway and mechanism of GPX4 inactivation.

**Figure S4:** Sevoflurane‐induced disruption of iron homeostasis and activation of ferroptosis.

**Figure S5:** Bridging mechanisms linking key ferroptosis regulators to synaptic impairment.

**Figure S6:** Integrated schematic of ferroptosis, synaptic dysfunction, and cognitive phenotype.

**Figure S7:** Comparative analysis of representative nanocarrier types and their brain‐targeting capabilities.

**Figure S8:** Drug release mechanisms of ROS/pH/enzyme‐responsive nanosystems.

**Figure S9:** Schematic illustration of the three‐tier evaluation indices commonly used in ferroptosis‐intervention studies.

**Table S1:** Overview of sevoflurane‐associated mechanisms of synaptic injury and cognitive impairment.
**Table S2:** Relationship between key ferroptosis molecules and synaptic dysfunction.
**Table S3:** Summary of methodological heterogeneity in ferroptosis biomarker detection.
**Table S4:** Comparison of nanodrug delivery system types and their characteristics.
**Table S5:** Timeline and regulatory milestones for clinical translation of targeted ferroptosis interventions via nanodelivery systems.

## Data Availability

The data that support the findings of this study are available from the corresponding author upon reasonable request.
