# Supplementary figures and images for: Targeting Ferroptosis Pathways for Synaptic Protection in Sevoflurane‐Induced Cognitive Impairment: A Nanomedicine Approach
Source: CNS Neurosci Ther. 2026 Mar 31;32(4):e70850. doi: 10.1002/cns.70850 (PMC13140902; doi:10.1002/cns.70850)

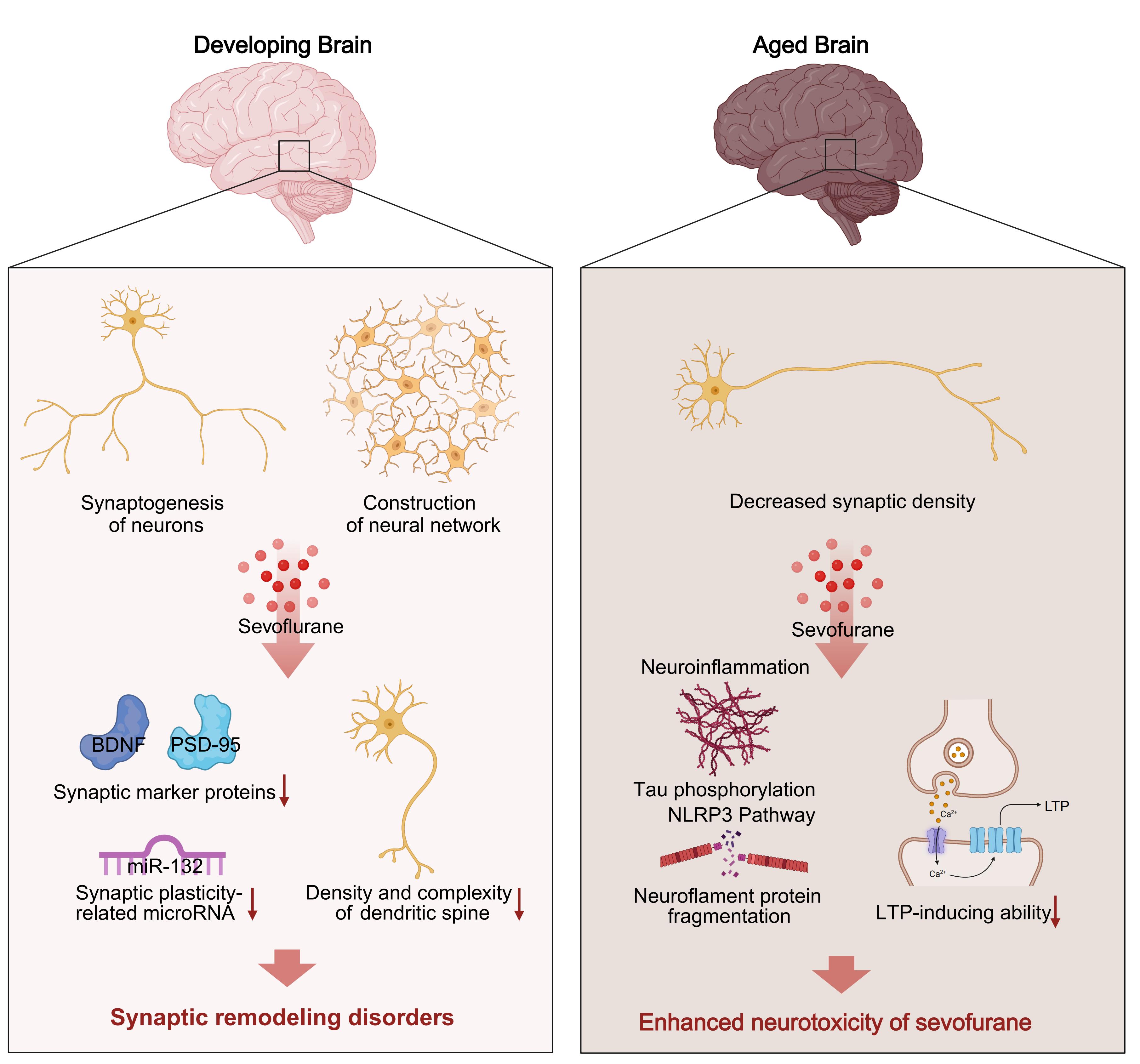

Supplement: Supplementary file 1 — Figure S1: Mechanistic illustration of synaptic plasticity vulnerability during developmental and aging stages. [file CNS-32-e70850-s009.jpg]

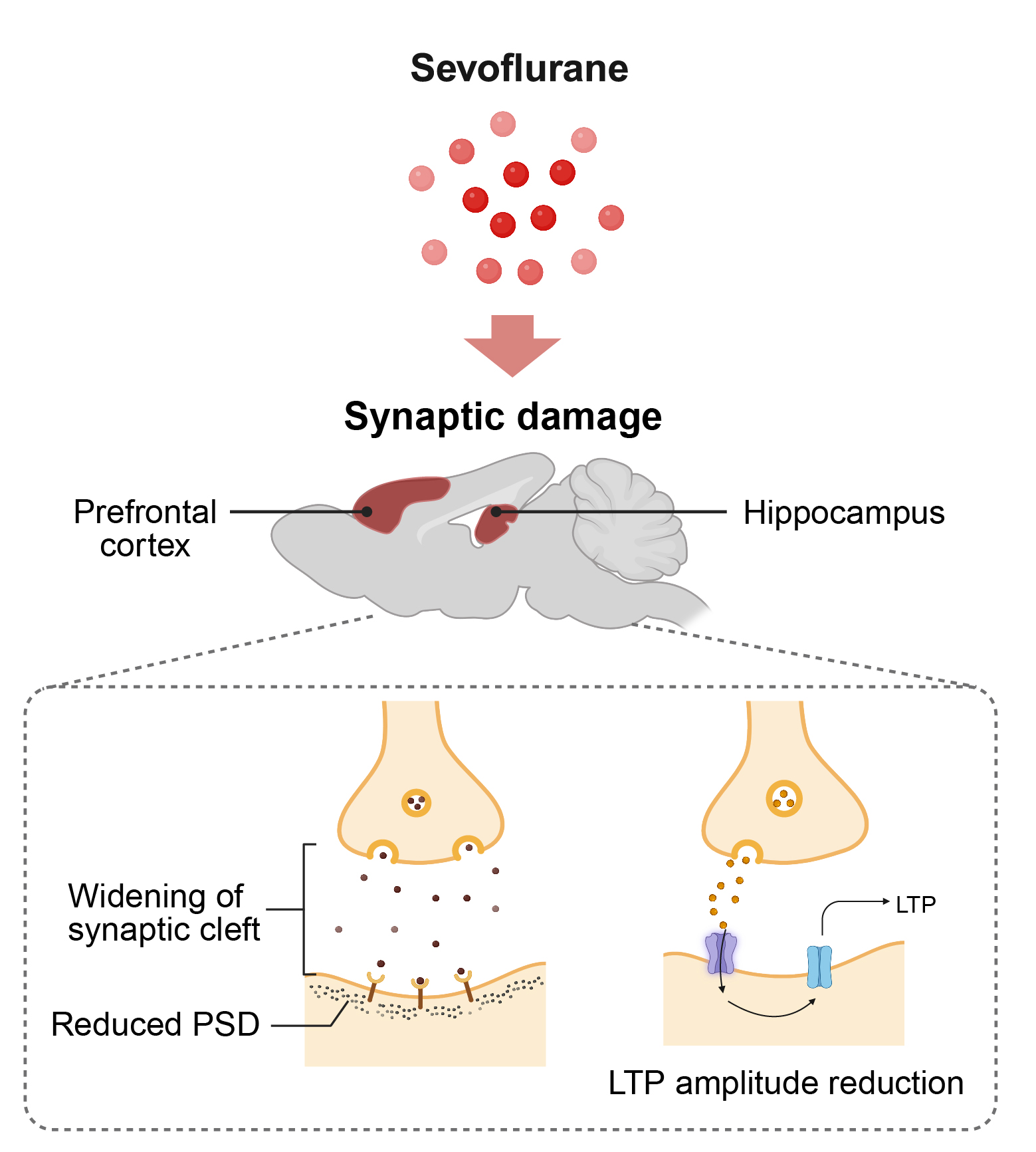

Supplement: Supplementary file 2 — Figure S2: Mechanistic illustration of regional brain dysfunction induced by sevoflurane‐related POCD. [file CNS-32-e70850-s008.jpg]

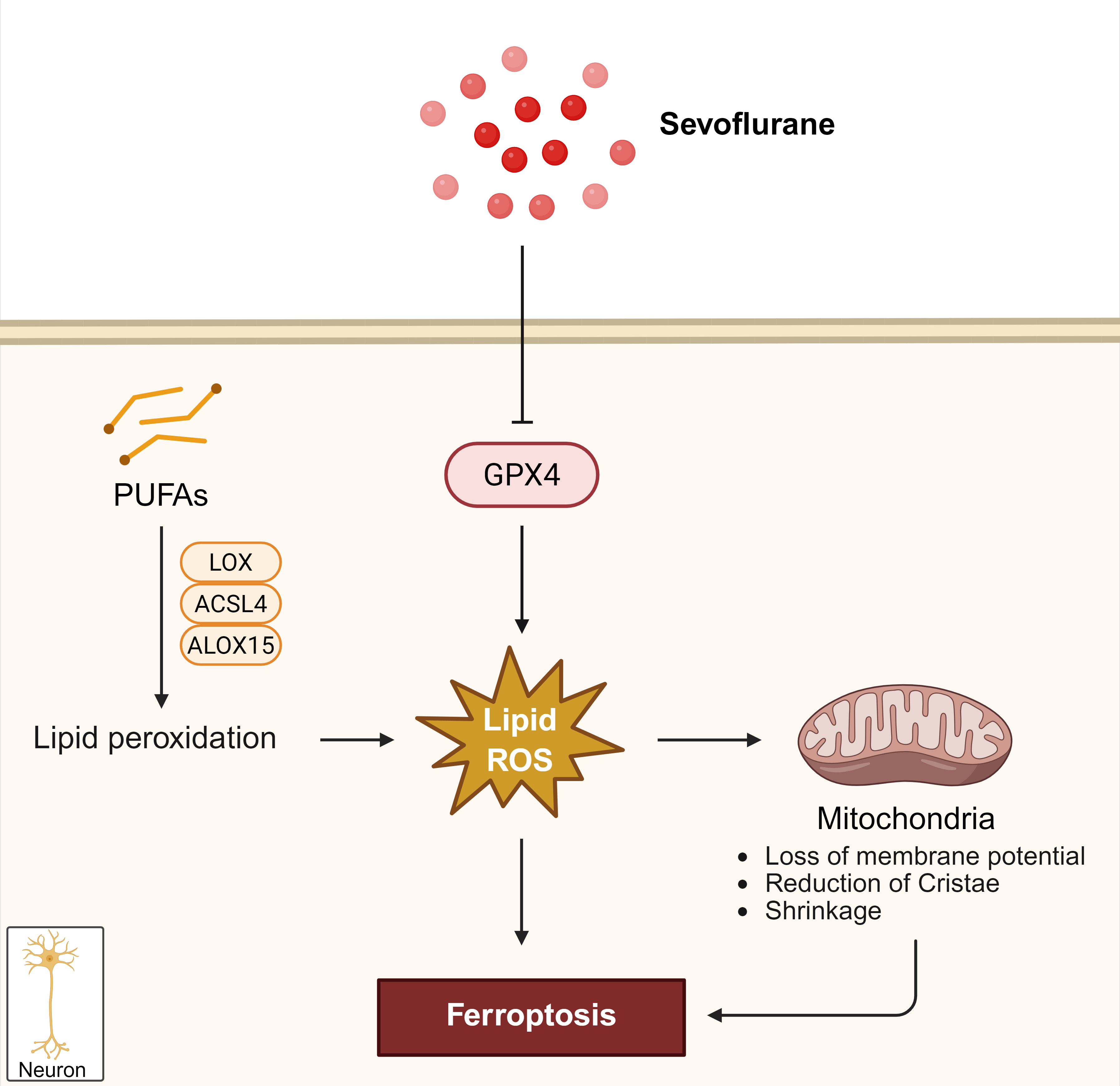

Supplement: Supplementary file 3 — Figure S3: Canonical ferroptosis pathway and mechanism of GPX4 inactivation. [file CNS-32-e70850-s006.jpeg]

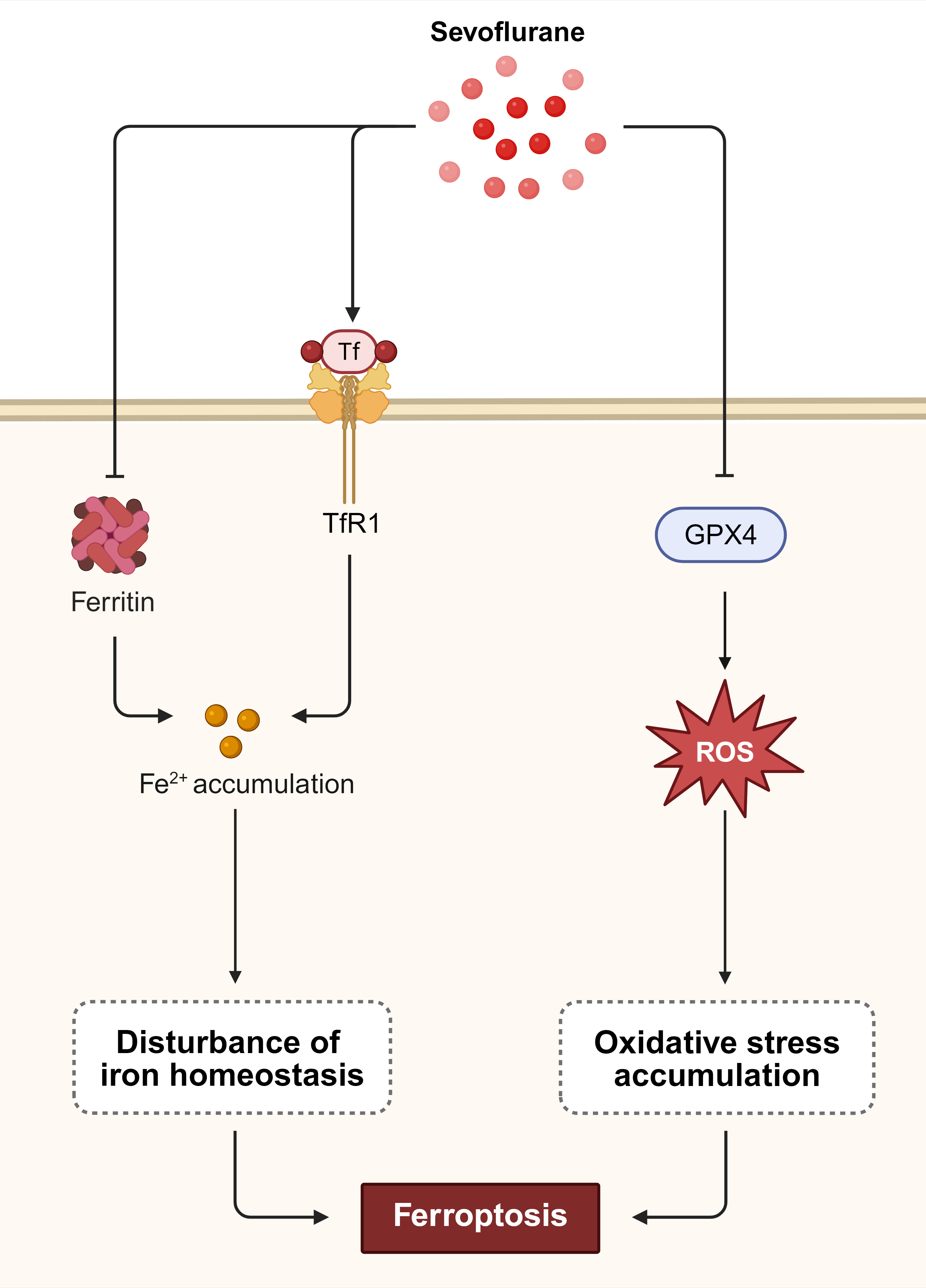

Supplement: Supplementary file 4 — Figure S4: Sevoflurane‐induced disruption of iron homeostasis and activation of ferroptosis. [file CNS-32-e70850-s002.jpeg]

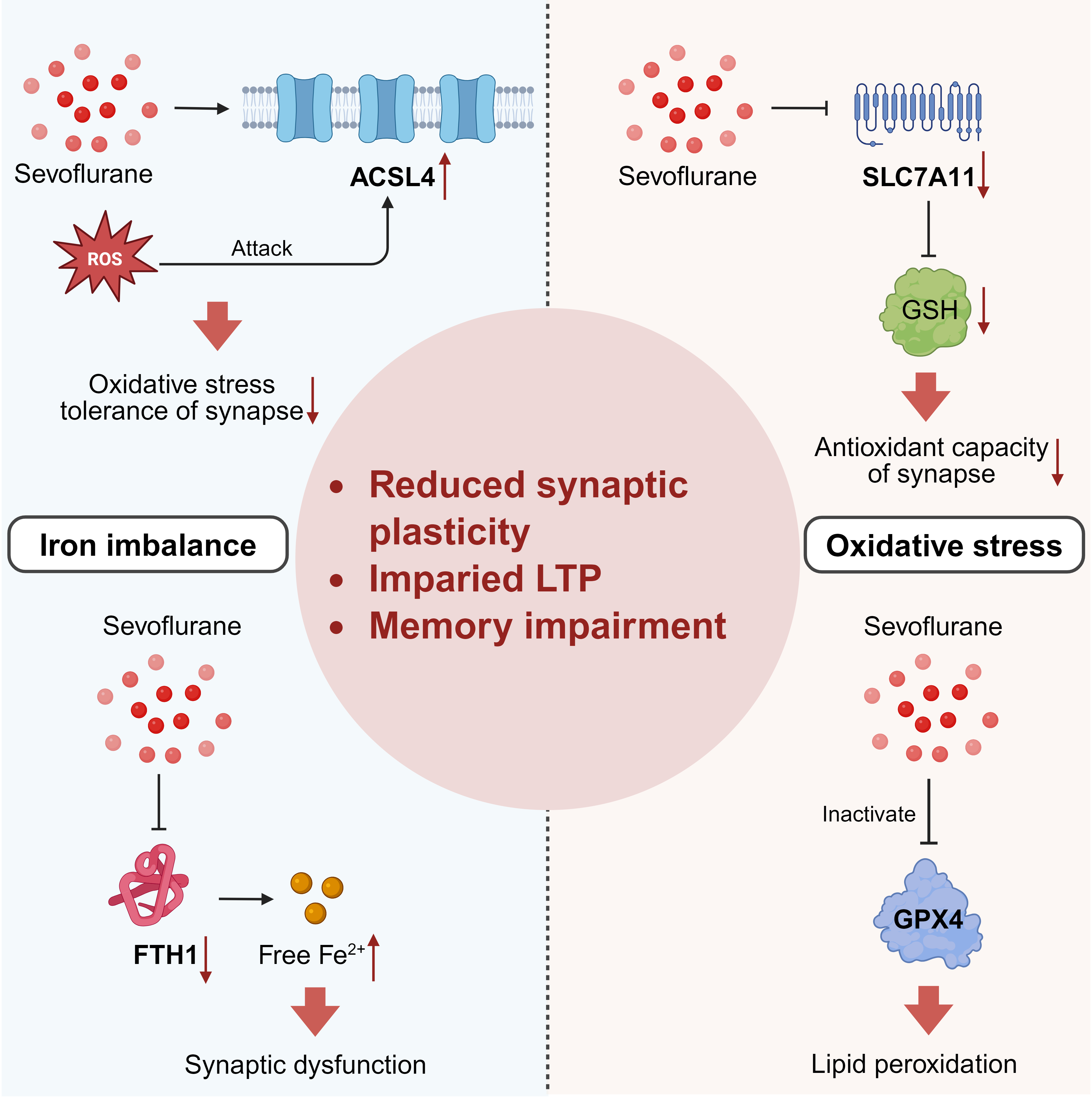

Supplement: Supplementary file 5 — Figure S5: Bridging mechanisms linking key ferroptosis regulators to synaptic impairment. [file CNS-32-e70850-s003.jpeg]

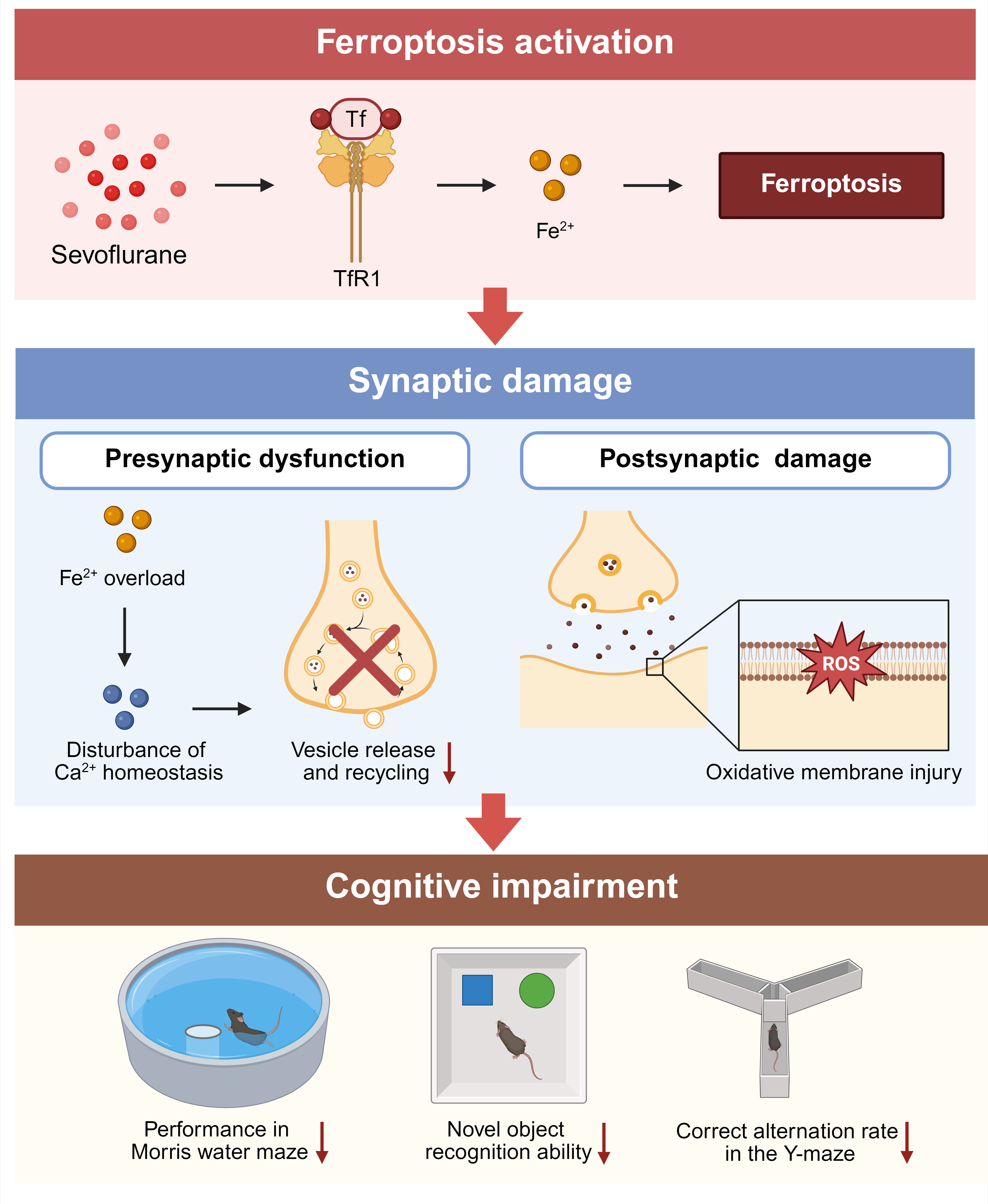

Supplement: Supplementary file 6 — Figure S6: Integrated schematic of ferroptosis, synaptic dysfunction, and cognitive phenotype. [file CNS-32-e70850-s005.jpeg]

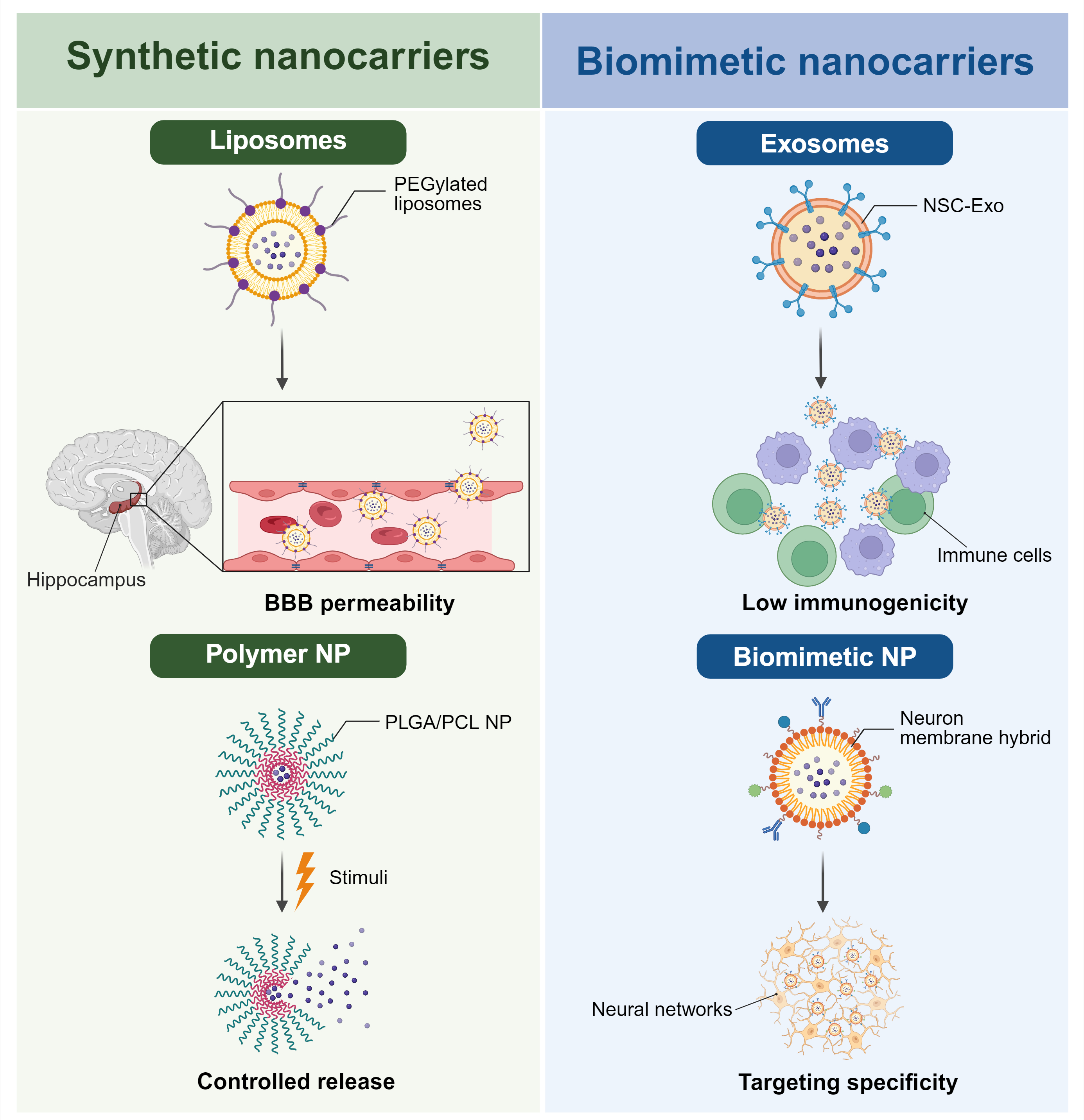

Supplement: Supplementary file 7 — Figure S7: Comparative analysis of representative nanocarrier types and their brain‐targeting capabilities. [file CNS-32-e70850-s010.jpeg]

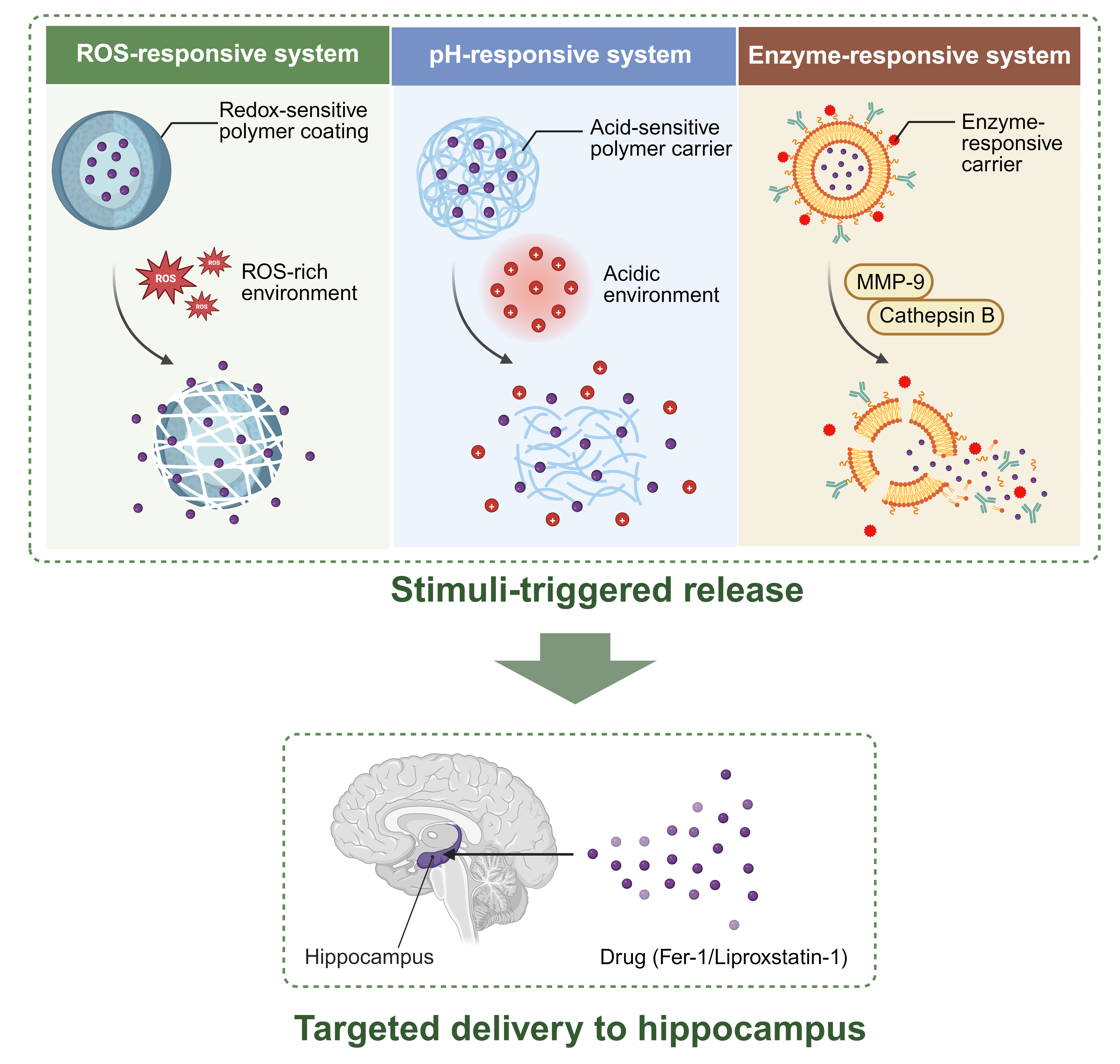

Supplement: Supplementary file 8 — Figure S8: Drug release mechanisms of ROS/pH/enzyme‐responsive nanosystems. [file CNS-32-e70850-s001.jpeg]

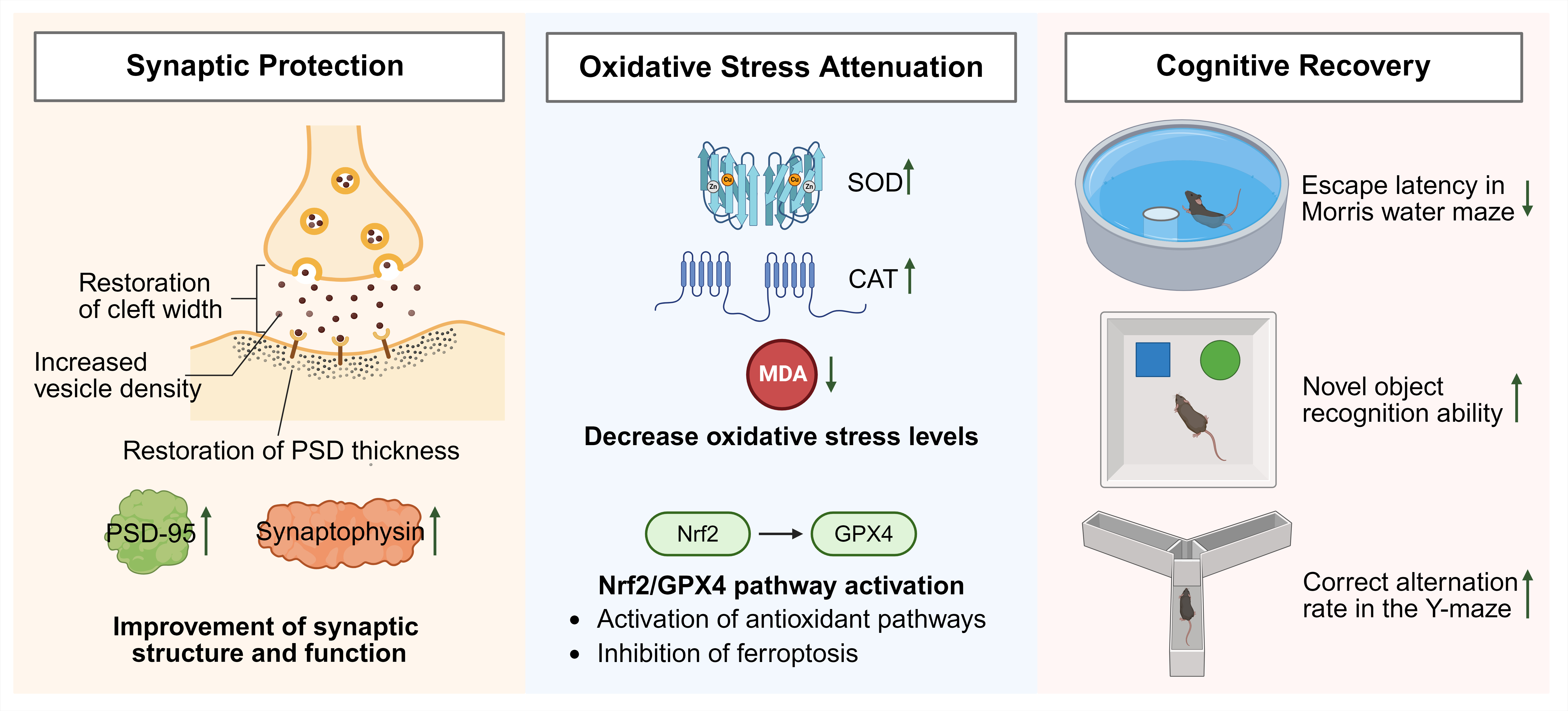

Supplement: Supplementary file 9 — Figure S9: Schematic illustration of the three‐tier evaluation indices commonly used in ferroptosis‐intervention studies. [file CNS-32-e70850-s004.jpeg]
