# Supplementary material for: Targeting Ferroptosis Pathways for Synaptic Protection in Sevoflurane‐Induced Cognitive Impairment: A Nanomedicine Approach
Source: CNS Neurosci Ther. 2026 Mar 31;32(4):e70850. doi: 10.1002/cns.70850 (PMC13140902; doi:10.1002/cns.70850)
Supplement: Supplementary file 10 — Table S1: Overview of sevoflurane‐associated mechanisms of synaptic injury and cognitive impairment. Table S2: Relationship between key ferroptosis molecules and synaptic dysfunction. Table S3: Summary of methodological heterogeneity in ferroptosis biomarker detection. Table S4: Comparison of nanodrug delivery system types and their characteristics. Table S5: Timeline and regulatory milestones for clinical translation of targeted ferroptosis interventions via nanodelivery systems. [file CNS-32-e70850-s007.docx]

**Table S1. Overview of Sevoflurane-Associated Mechanisms of Synaptic Injury and Cognitive Impairment.**

| **Mechanism (PMID)** | **Key Molecules / Events** | **Impact on Synapses** |
| --- | --- | --- |
| Oxidative Stress  [6, 20] | Excessive ROS accumulation; suppression of Nrf2/ARE signaling; NEAT1 upregulation; inhibition of antioxidant enzymes (HO-1, GPX4) | Lipid peroxidation of synaptic membranes, mitochondrial oxidative damage, impaired neurotransmission |
| Neuroinflammation / Microglial Activation  [21, 64] | Microglial M1 polarization (↑iNOS, IL-1β, TNF-α; ↓ARG1); altered Iba-1 expression; inflammatory cytokine release | Disruption of synaptic remodeling; reduced synaptic repair capacity; interference with neurotrophic signaling (BDNF-TrkB) |
| Downregulation of Synaptic Structural Proteins  [163, 164] | Reduced expression of β-neurexin, neuroligin-1, PSD-95, and NMDA receptor subunits NR2A/NR2B | Loss of excitatory synaptic stability; altered excitatory/inhibitory balance within hippocampal circuits |
| Impaired Synaptic Plasticity (LTP/AP Deficits)  [3, 165] | Attenuated hippocampal LTP; reduced evoked action potential firing; decreased expression of BDNF, Arc, Egr1 | Decreased synaptic strength and signal integration, leading to learning and memory impairment |
| Mitochondrial Dysfunction & Ferroptosis  [6, 164] | Iron accumulation; mitochondrial ROS and lipid hydroperoxide elevation; altered ferroptosis markers (ACSL4, GPX4, SLC7A11); PLIN4-Hippo pathway activation | Energy failure (ATP↓); mitochondrial membrane potential loss; synaptic degeneration and neuronal apoptosis |
| Calcium Dyshomeostasis and Membrane Excitability  [22, 46] | Ca²⁺ overload; mitochondrial permeability transition pore (mPTP) opening; reduced mitochondrial respiratory activity | Abnormal presynaptic vesicle release, dysregulated postsynaptic potentials, impaired network excitability |
| Network-Level Connectivity Disruption  [24, 25] | Reduced hippocampo-cortical functional connectivity; suppressed default mode network (DMN) dynamics; loss of synchronized FC states | Impaired long-range synaptic integration; network fragmentation underlying postoperative cognitive dysfunction |

**Table S2. Relationship Between Key Ferroptosis Molecules and Synaptic Dysfunction.**

| **Molecule (PMID)** | **Mechanism of Action in Ferroptosis** | **Potential Impact on Synaptic Function** |
| --- | --- | --- |
| GPX4 [10, 42] | Major antioxidant enzyme that detoxifies lipid peroxides and preserves phospholipid membrane integrity | Loss of GPX4 facilitates lipid peroxidation, increases oxidative vulnerability of synaptic membranes, and may predispose to synaptic transmission deficits |
| SLC7A11 [10, 56] | Mediates cystine uptake to support glutathione (GSH) synthesis and maintain redox homeostasis | Reduced SLC7A11 limits GSH production and may weaken antioxidant defense at synapses, increasing oxidative susceptibility |
| ACSL4 [8, 46] | Catalyzes esterification of polyunsaturated fatty acids (PUFAs), generating oxidizable phospholipid substrates | ACSL4 upregulation enhances vulnerability of synaptic membranes to lipid peroxidation and may contribute to structural instability of excitatory synapses |
| FTH1 [51, 57] | Ferritin heavy chain stores Fe²⁺ to buffer the labile iron pool and mitigate iron toxicity | Reduced ferritin expression expands labile iron availability and may aggravate oxidative stress at presynaptic terminals |
| ALOX15 [41, 48] | Lipoxygenase enzyme promoting enzymatic lipid peroxidation and lipid hydroperoxide formation | ALOX15 activation may accelerate membrane oxidative damage and compromise synaptic membrane function |
| TfR1 [10, 51] | Increases neuronal iron uptake and elevates intracellular iron burden | Elevated TfR1 may contribute to iron overload and oxidative vulnerability at synaptic sites |
| FPN1 [41, 51] | Ferroportin regulates iron efflux and controls intracellular iron export | Downregulation of FPN1 may cause iron retention and increase oxidative injury in synaptic regions |
| LOX [41, 48] | Catalyzes PUFA oxidation and generates reactive lipid species | LOX activation may promote lipid peroxidation and predispose synapses to structural degeneration |
| NRF2 [20, 166] | Master antioxidant regulator activating HO-1, NQO1 and other defense genes | NRF2 suppression may impair antioxidant capacity and increase synaptic susceptibility to oxidative stress |

**Table S3. Summary of Methodological Heterogeneity in Ferroptosis Biomarker Detection.**

| **References (PMID)** | **Ferroptosis Biomarkers Detected** | **Detection Methods** | **Criteria for “Significant Abnormality”** | **Sample Type** | **Basis for Core Conclusions** |
| --- | --- | --- | --- | --- | --- |
| PMID: 37874483 | Fe²⁺; H₂O₂; NOX4; GPX4; SLC7A11; TfR; TF; FPN; MDA | qRT-PCR; Western Blot; FerroOrange; BODIPY 581/591; DCFH-DA fluorescence | Significant increase in iron uptake markers and lipid peroxidation, with concurrent reduction of GPX4/SLC7A11 (P < 0.05) | Mouse hippocampal neurons and brain tissue | Sevoflurane induced ferroptosis via ER stress–ATF3 activation, promoting iron overload, ROS accumulation, and suppression of antioxidant defenses |
| PMID: 40378436 | TfR; FPN; NOX4; ALOX12; ALOX15; SLC7A11; GPX4 | Western Blot; qRT-PCR; immunofluorescence | Upregulation of iron transport and lipid oxidation enzymes with GPX4 suppression (P < 0.05) | HT22 cells; primary hippocampal neurons | Ferroptosis was triggered through ATM/p53 and JNK/p38 MAPK pathways, linking DNA damage to iron accumulation and lipid peroxidation |
| PMID: 34734006 | ACSL4; GPX4; SLC7A11; Fe²⁺; MDA; 4-HNE | Western Blot; qRT-PCR; MDA assay | Increased ACSL4 and decreased GPX4/SLC7A11 relative to control (P < 0.05) | SH-SY5Y neuronal cells | ACSL4 knockdown protected against sevoflurane-induced ferroptosis by restoring antioxidant function and mitochondrial integrity |
| PMID: 35935755 | Mitochondrial ROS; Fe²⁺; mPTP opening; ΔΨm; Mito-lipid hydroperoxides | MitoSOX; JC-1; Fluo-4; Prussian blue staining; Western Blot | Significant elevation of mitochondrial ROS and iron staining with decreased mitochondrial membrane potential (P < 0.05) | Mouse hippocampal tissue; primary neurons | Sevoflurane exposure induced mitochondrial iron overload and dysfunction, driving ferroptosis and cognitive deficits |
| PMID: 38928480 | GPX4; miR-182-5p; Fe²⁺; lipid peroxides | WB; qRT-PCR; FerroOrange; MitoSOX; BODIPY 581/591 | Reduced GPX4 and increased mitochondrial Fe²⁺ and lipid peroxidation relative to untreated controls (P < 0.05) | Mouse cochlear hair cell synapses | Ferroptosis inhibition preserved ribbon synapses and prevented sevoflurane-induced auditory dysfunction |
| PMID: 33539795 | Ferritin (FTH1); TfR; FPN; MDA; ROS | μ-XRF (synchrotron micro–X-ray fluorescence); WB | Elevated ferritin and decreased TfR/FPN indicating disrupted iron efflux and oxidative damage (P < 0.05) | Mouse hippocampus and cortex | Sevoflurane induced iron accumulation via FPN/hepcidin pathway, leading to oxidative stress and cognitive decline |
| PMID: 40046685 | GPX4; ACSL4; MDA | Western Blot; TBA method | GPX4 recovery and decreased MDA after Liproxstatin-1 treatment (P < 0.05) | Mouse hippocampal tissue (nano-intervention model) | Lipid peroxidation inhibitor Lip-1 reversed sevoflurane-induced ferroptosis and improved cognitive performance |

Table Notes:

1. “Criteria for Significant Abnormality” refer to statistically significant changes relative to the control group (P < 0.05), in accordance with methodological conventions in ferroptosis research [8, 10].
2. Abbreviations for detection methods: WB = Western Blot, qPCR = Quantitative Real-Time PCR, TBA = Thiobarbituric Acid Assay, μ-XRF = Micro–X-Ray Fluorescence Imaging, IF = Immunofluorescence.
3. Fluorescent probes used in the cited studies include MitoSOX (mitochondrial ROS), BODIPY 581/591 (lipid peroxidation), and FerroOrange/FeRhoNox-1 (free Fe²⁺).
4. “Synaptosomes” in the sample type refer to synapse-enriched tissue fractions isolated by density gradient centrifugation for targeted ferroptosis marker detection.

**Table S4. Comparison of Nanodrug Delivery System Types and Their Characteristics.**

| **System Type (PMID)** | **Representative Carrier** | **Delivery Advantages** | **Limitations and Challenges** |
| --- | --- | --- | --- |
| Liposomes [12, 87] | RVG-modified liposomes encapsulating Ferrostatin-1 (Fer-1) | Excellent encapsulation efficiency, enhanced BBB penetration via ligand modification, and tunable release kinetics | Poor long-term stability, potential leakage during storage, and batch-to-batch variability |
| Polymeric Nanoparticles [88, 89] | PLGA nanoparticles loaded with Liproxstatin-1 | Controlled release profile, favorable biodegradability, and improved pharmacokinetics in the CNS | Complex chemical synthesis, uncertain long-term toxicity, and scalability limitations |
| Exosome-Based Systems [90, 91] | Exosome-encapsulated ferroptosis inhibitors (Fer-1 / Lip-1) | Strong BBB penetration, natural membrane composition, and low immunogenicity | Difficult purification, low yield, and heterogeneity in exosome cargo loading |
| Biomimetic Membrane Systems [86, 92] | Neuron-membrane–coated PLGA nanoparticles | High targeting specificity, homotypic recognition, and enhanced immune evasion | Surface modification complexity and limited reproducibility of membrane coating efficiency |
| Composite Core–Shell Systems [12, 109] | PLGA core + lipid shell + RVG ligand | Multifunctional assembly enabling precise brain targeting and enhanced BBB transport | Structural complexity, high material synthesis cost, and difficulty in large-scale production |
| Micelle Systems [167] | PEG–PLA micelles encapsulating Fer-1 | High stability in circulation, suitable for hydrophobic ferroptosis inhibitors, and improved brain accumulation | Prone to dissociation in plasma and potential micelle aggregation |
| ROS-Responsive Nanocarriers [114] | ROS-triggered polymeric nanoparticles for ferroptosis inhibition | Pathological ROS-specific activation, lesion-triggered drug release, and reduced systemic toxicity | Limited synthetic stability and difficulties in fine-tuning ROS sensitivity in vivo |
| Magnetic Targeting Systems [11, 101] | Fe₃O₄ magnetic nanoparticles loaded with Liproxstatin-1 | Magnetically guided targeting, external control capabilities, and improved spatial localization of ferroptosis inhibitors | Safety of magnetic materials requires further evaluation and potential chronic neurotoxicity |

**Table Notes:**

1. “Representative Carrier” refers to nanoplatforms reported in peer-reviewed studies of ferroptosis-targeted CNS drug delivery [12, 88].
2. BBB penetration advantages were determined based on in vitro BBB models or in vivo brain distribution experiments as described in the referenced studies.
3. Limitations summarize methodological challenges of nanoparticle fabrication, cargo stability, immunogenicity, and long-term biosafety reported in nanomedicine literature.

**Table S5. Timeline and Regulatory Milestones for Clinical Translation of Targeted Ferroptosis Interventions via Nanodelivery Systems.**

| **Translation Phase** | **Time Span** | **Core Regulatory Milestones** | **Key Validation Studies** | **Supporting Literature (PMID)** |
| --- | --- | --- | --- | --- |
| Preclinical Validation | 2-3 years | Completion of long-term toxicity studies in non-human primates and validation in humanized models | Synaptic repair experiments in brain organoids, metabolic studies in large animals | 37242612, 38030651 |
| IND Application + Phase I Clinical Trial | 1-2 years | IND approval, single/multiple-dose safety verification | PK/PD studies in healthy volunteers, PET brain distribution imaging | 35710426, 38027994 |
| Phase II Clinical Trial | 2-3 years | Preliminary efficacy confirmation, dose exploration | Randomized controlled trial (MoCA Score), subgroup analysis | 39735960, 38619150 |
| Phase III Clinical Trial | 3-4 years | Multicenter efficacy confirmation, production process validation | Biomarker-imaging correlation studies, concomitant medication assessment | 36068571, 35710426 |
| Marketing Approval + Phase IV Monitoring | 1-2 years | NDA/BLA approval, post-marketing long-term safety monitoring | 10-year accumulation risk assessment, adverse reaction registration system | 38030651, 36954621 |

Notes: IND = Investigational New Drug; PK = Pharmacokinetics; PD = Pharmacodynamics; MoCA = Montreal Cognitive Assessment; NDA = New Drug Application; BLA = Biologics License Application.
